# Women’s experiences and perceptions of anxiety and stress during the perinatal period: a systematic review and qualitative evidence synthesis

**DOI:** 10.1186/s12884-021-04271-w

**Published:** 2021-12-06

**Authors:** Megan McCarthy, Catherine Houghton, Karen Matvienko-Sikar

**Affiliations:** 1 School of Public Health, University College Cork, Cork, Ireland; 2 School of Nursing and Midwifery, National University of Ireland Galway, Galway, Ireland

**Keywords:** Stress, Anxiety, Pregnancy, Postpartum, Perinatal, Qualitative Evidence Synthesis

## Abstract

**Background:**

The perinatal period, from pregnancy to the first year postpartum, is a transitional period that can result in anxiety and stress for some women. Perinatal anxiety and stress can adversely impact the physical and psychological health of women and children. Understanding women’s lived experiences of perinatal anxiety and stress is essential to better support women. The aim of this qualitative evidence synthesis was to examine women’s experiences and perceptions of, and barriers and facilitators to coping with, perinatal anxiety and stress.

**Methods:**

Databases CINAHL, EMBASE, MEDLINE, PsycINFO and Maternity and Infant Care were searched from inception to June 2020. Eligible studies included women who were pregnant or up to one year postpartum and examined women’s experiences of anxiety and/or stress during the perinatal period. Data were synthesised using thematic synthesis.

**Results:**

Of 20,318 identified articles, 13 studies met inclusion criteria and were included in this review. Five key themes emerged: Social support, women’s experiences of healthcare, social norms and expectations, factors that impact on coping and mother and baby’s health.

**Conclusion:**

This review provided a comprehensive synthesis of perinatal anxiety and stress. Findings indicate that increased support for perinatal mental health in antenatal and postpartum care is needed. Addressing unrealistic expectations and conceptualisations of motherhood is also important to better support women. Enhancing women’s social support networks and provision of clear and consistent information are also essential to support women and minimise stress and anxiety in the perinatal period.

**Supplementary Information:**

The online version contains supplementary material available at 10.1186/s12884-021-04271-w.

## Background

The perinatal period, defined here as the period from pregnancy to the first year postpartum, is a time of transition, including profound changes that can lead to anxiety and stress for some women [34, 39]. Perinatal anxiety and stress are highly correlated through distinct constructs, that can result from low material resources, poor social support [53], work/family responsibilities [44], and pregnancy complications [5]. Perinatal anxiety affects approximately 17% of women [20]; while up to 84% of women experience perinatal stress [56]. Perinatal anxiety and stress can negatively impact women and children’s health ([34]; perinatal anxiety and stress are associated with increased risk of preeclampsia, miscarriage, low infant birth weight, and preterm delivery [16, 20]. Perinatal anxiety and/or stress are also associated with maternal behaviours such as alcohol consumption [54], breastfeeding [17], and smoking [42]. Adverse child outcomes include increased risk of poor cardiovascular health [38], obesity [47], self- regulation and neurodevelopmental difficulties [51].

Given the adverse consequences perinatal anxiety and stress has for maternal and child outcomes, supporting women during this period is essential. Effects of interventions designed to target anxiety and/or stress during the perinatal period are inconsistent however [29, 33, 35]; this inconsistency, coupled with the multiple potential sources of perinatal anxiety and stress, highlights the need to better understand women’s experiences of perinatal anxiety and stress [45]. Research on anxiety and stress in the perinatal period has been largely quantitative to date however, with limited qualitative research exploring women’s lived experiences of perinatal anxiety and stress [13, 21]. Understanding women’s lived experiences of anxiety and stress is essential to inform the development and delivery of effective interventions to help women cope with perinatal anxiety and/or stress [45]. To date, one qualitative evidence synthesis (QES) of women’s experience of psychological distress has been conducted [45]. This review focused on pregnancy only, thus missing the longitudinal nature of anxiety and stress during the transitional perinatal period.

The aim of this QES was to comprehensively explore women’s lived experiences and perceptions of anxiety and stress across the perinatal period and to examine coping strategies for perinatal anxiety and stress.

## Methods

The review protocol was registered on the PROSPERO registry (CRD42020193757). The PRISMA and ENTREQ statements guided the review conduct and reporting [37, 50].

### Criteria for considering studies for this review

#### Types of studies

We included studies that used qualitative data collection and analysis methods. Mixed methods studies were only included if the qualitative data collection and analysis were adequately described in the studies, and if the findings and interpretations were provided at a sufficient depth. There were no restrictions based on language.

#### Search methods

The following electronic databases were searched in July 2020: CINAHL, EMBASE, MEDLINE, PsycINFO and Maternity and Infant Care (See Table 1 for search terms used).

**Table 1:** Search terms

	Perinatal OR antepartum OR antenatal OR prenatal OR postpartum OR postnatal OR pregnancy OR pregnant OR mother* OR mom OR mum OR maternal
AND	stress* OR distress OR anxiety
AND	qualitative OR interview* OR ‘focus group’ OR ethno* OR theme OR thematic OR narrative OR ‘action research’

#### Selection of studies

Titles and abstract screening, and full text screening were conducted in duplicate (blinded); any discrepancies were resolved by consensus discussion between reviewers. Data were extracted using a standardised data collection form (See Supplementary file 1).

#### Assessment of methodological limitations

The critical appraisal skills programme (CASP) was used to assess methodological limitations of individual studies [31]. One reviewer (blinded) conducted assessment; half of all appraisals were checked by a second reviewer (blinded), with full agreement.

#### Data synthesis

The RETREAT criteria (Review question, Epistemology, Time/Timeframe, Resources, Expertise, Audience & Purpose, Type of Data) were used to consider the appropriate synthesis methodology [8]. Thematic synthesis was chosen to allow for a transparent and inductive synthesis of primary studies [48]. Data analysis was managed using QSR NVIVO. Initial line-by-line coding was conducted by (blinded). This was followed by evaluating all codes to determine consistency of interpretation at the line-by-line coding stage [48]. Descriptive themes were subsequently developed and were applied to the review aims and questions. Identification and evaluation of similarities and differences across descriptive themes informed development of analytic themes [48]. (blinded) independently conducted all stages of synthesis with support from (blinded) and (blinded).

#### Assessment of confidence in the findings

The GRADE CERQual (Confidence In The Evidence From Reviews Of Qualitative Research) approach was used to assess the confidence in findings [30]. Confidence in all study findings was assessed by one reviewer (blinded) using the GRADE CERQual approach, with one third of the review findings crosschecked by (blinded).

## Results

**Fig. 1:**
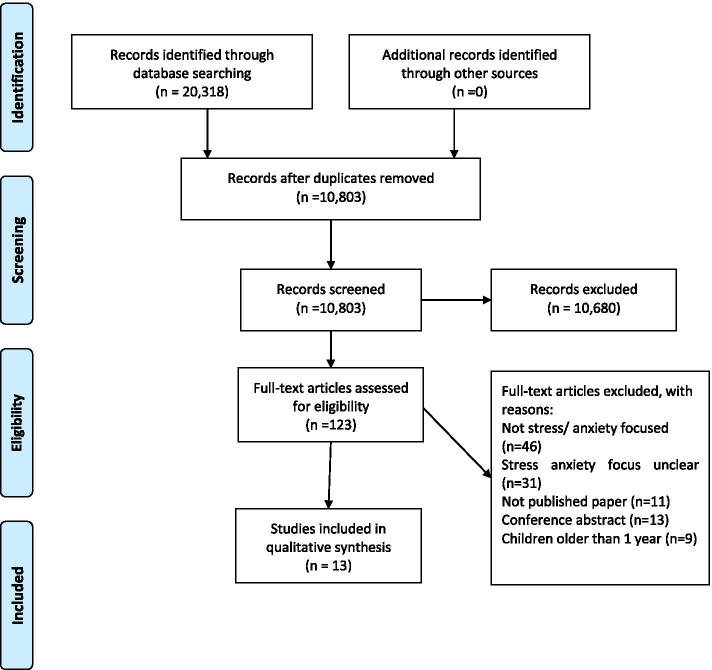
Prisma Flow Diagram

Thirteen studies met inclusion criteria (see Figure 1). Characteristics of included studies are summarised in Table 2. One study was assessed as having no methodological limitations and 12 studies were assessed as having minor methodological limitations. See Supplementary File 2 for full details of the assessment of methodological limitations for each study.

**Table 2:** Characteristics of included studies

Study	Country	Participants	SES and Ethnicity	Study aim	Data collection	Analytic Approach
[1]	USA	Pregnant and postpartum women	SES: Filipino and Hawaiian (Low SES), Japanese (middle to upper SES) Mixed Ethnicities	To describe themes of stressors reported by women living in rural communities known as East Hawaii on the island of Hawaii	Focus groups	Ethnographic summary approach and systematic coding via content analysis
[2]	Pakistan	Pregnant women (n=28)	Different SES and Ethnic groups	This study aimed to explore components and dimensions of pregnancy anxiety.	Semi-structured interviews	Content analysis
[3]	England	Pregnant women (n=19)	SES: Low-income urban, peri-urban, and rural populations Ethnicity: not given	The study aimed to develop a culturally appropriate, feasible, and acceptable psychological intervention for perinatal anxiety in the context of a low-income population in Pakistan.	Interviews with pregnant women attending the outpatient clinics	Framework analysis
[4]	England	Women 6-12 weeks after birth (n=148)	SES: 43.2% were educated to degree level and only two women had no educational qualifications (1.4%). Most were in employment (83%) Ethnicity: White European (94.3%), followed by Asian (2.8%), other (1.9%), and African (0.9%)	The study aimed to address these methodological issues by identifying key stressors during the perinatal period, using a method that encourages open and honest reporting.	Women wrote anonymously about a situation they found stressful as part of the Health after Birth Trial (HABiT) of expressive writing	Content analysis
[7]	USA	Pregnant women (n=23)	Ethnicity: White (91.1) Black (8.7) SES: Low income mothers	The study aimed to describe stress exposures, stress responses, and priorities for stress reduction among a sample of low-income rural pregnant women	Qualitative interviews	Qualitative descriptive approach
[11]	USA	Pregnant women (n=96)	Ethnicity: White (52) African Americans (44) SES: Low income	The study aimed to identify factors that influenced stress, healthy lifestyle behaviors (healthful eating and physical activity) during pregnancy	Focus group discussions	-
[14]	USA	Mothers who birthed an infant aged between 6 weeks and 6 months (n=21)	Ethnicity: Caucasian (95%) African American (5%) SES: Low income mothers	This study aimed to determine how maternal distress influences mothers’ transition to becoming a mother and to validate the use of the Maternal Distress Concept in the clinical setting	Semi-structured interviews	Directed content analysis
[19]	England	Women who gave birth within 9 months postpartum (n=19)	Not given	The aim of the study was to explore women’s experience of anxiety in pregnancy and their views on the use of anxiety instruments in antenatal care.	Focus group discussions	Template analysis
[24]	England	Women/pregnant women within one year postpartum (n=23)	Mixed Ethnicities SES not given	The aim of the current study is to use a qualitative approach to explore women’s experience of PNA, in particular, considering the main sources of their anxiety (i.e. triggers) and the support/coping strategies they use in both online and offline contexts. the aim of this study is twofold. First, to qualitatively explore women’s experience of anxiety triggers and support in the perinatal period; and second to gain insight into what online support is acceptable for women with PNA.	Focus groups	Inductive thematic analysis
Razurel et al., [41]	Switzerland	Women six weeks after birth (n=90)	Mixed SES Ethnicity not given	The aims of this study were to investigate events perceived as stressful by primiparous mothers during the postpartum period and perceived social support, and to identify coping strategies.	Semi- structured interviews	An iterative approach was used to construct an ‘analytical tree'
[43]	Australia	Mothers of infants up to one year (n=20)	SES: Participants were on average more socioeconomically-advantaged than the general population of women who have recently given birth in Victoria Ethnicity not given	This study aimed to investigate the sources of worry and anxiety that women identify in the perinatal social and health milieu, the language and contexts they use to describe them, and the meaning that they ascribe to their experiences.	Discussion groups	Thematic analysis
[26]	Tanzania	Pregnant and postpartum women (n=10)	Mixed SES Ethnicity not given	To explore and understand the experiences and priorities of pregnant women living with fears and worries related to fetal/infant and maternal health, the birthing process and ability to parent the infant (ie,pregnancy-related anxiety (PRA)) in Mwanza, Tanzania	Semi-structured interviews	Descriptive phenomenological approach
[46]	USA	Pregnant women between gestational weeks of 12 and 18 (n=31)	Ethnicity: White (74.1%) Asian (16.2%) and Black or African American (2.1%) SES: sample was highly educated with 85.5% having at least an associate’s degree, and 69.7% reported household incomes of $100,000	The aim of this study were to measure pregnancy-specific anxiety quantitatively and evaluate this anxiety qualitatively in women pregnant via IVF using a mixed methods approach by describing the level of pregnancy-related anxiety in women pregnant via IVF during early second trimester, and to identify themes in anxiety specific to pregnancy.	Semi structured interviews (open ended questions)	Content analysis

### Findings

Five themes, with 14 subthemes, of women’s experiences of perinatal anxiety and stress were identified (See Table 3 for Summary of themes and subthemes). From the findings presented here, key review findings (n=15) were developed and confidence in these findings were assessed using GRADE CERQual (See detailed assessments in Supplementary File 3).

**Table 3:** Themes and Subthemes

Theme (Studies themes identified in)	Descriptor	Subthemes (Studies subtheme identified in)
Social Support ([2] [3]; [4] [1]; ;[11]; [14] [26]; ;[19] [24] [7]; ;;[41];)	This theme examines the impact of social support on anxiety and stress during both pregnancy and the postpartum period	• Partner Support [2–4, 11, 14, 26] • Peer Support [11, 19, 24] • Family Support [1, 3, 4, 7, 11, 14, 24, 41]
Women’s Experiences of Health Care ([1] [2]; [3] [4]; ;[7]; [19] [24] [26]; ;;[41];)	This theme relates to the impact of women’s experiences of healthcare on perinatal anxiety and stress	• Perceived poor care from Healthcare Professionals ([1] [4]93 [2] [7, 19, 24, 26, 41];); • ;Access to healthcare services [2, 3, 19, 26] • Childbirth experiences [2–4, 19]
Factors that Impact on Coping ([1] [3] [7] [11]; ;;[19]; [24] [26] [41];);;	This theme examines some of the factors that may facilitate or hinder women’s coping during pregnancy and the postpartum period	• Behavioural Strategies (Affonso et al. 1993 [3, 7, 11, 19, 24, 26, 41];) • Faith [3, 7, 26] • Information [24, 43]
Social Norms and Expectations ([1] [3] [4] [11] [14]; ;;[;^24^] [26]; ;[41] [43] [19];);;	This theme related to women’s sense of pressure to adhere to perceived societal norms, which was a cause of stress and anxiety	• Being a “good mother” [4, 24, 41, 43] • Mental health stigma [3, 24, 43] • Role changes and responsibilities (Affonso et al. 1993 [3, 4, 11, 14, 24, 26, 43];)
Women’s and Baby’s Health ([2] [4]; [14] [26]; ;[43]; [46];)	This theme explored women’s experience of anxiety and stress in relation to their own health issues and the health status of their unborn or newly born baby	• Women’s health [14, 26, 46] • Baby’s health ([2] [4]; [43]; [46];)

### Theme one: Social Support

A consistent theme in 11 studies was the influence of social support on anxiety and stress during both pregnancy and the postpartum period (Table 3). Overall, women received different types of social support from peers, partners and families, including emotional, physical and informational support.

#### Partner support

In six studies, women discussed a lack of support from their partners and expressed a need for greater support (Table 3). Lack of partner support and poor communication was recognised as a key stressor by pregnant women: ‘*During that time (pregnancy) my husband was not around, he had travelled … and I needed him’* [26]. Women were often upset with their partners for not being present, helpful and understanding of their concerns [3, 4, 11, 14]. One woman reported how stressful and difficult it was being a mother without adequate support from her partner: ‘*At first it was hard because my boyfriend was scared of her and I didn’t have a lot of help. I had to do it all on my own’* [14].

#### Peer support

In three studies, women discussed the importance of peer support in reducing feelings of distress and anxiety (Table 3). Social support from other women who were or had been pregnant themselves was described as particularly helpful ‘*I think the biggest support and the biggest help people get is other mums that are going through exactly the same thing’* [24]. Women who had similar emotional experiences were seen as sources of reassurance and normalisation, which helped women feel confident, more tolerant of uncertainty, and less anxious and stressed [19, 24].

#### Family support

Family was another important source of support for women discussed by women in eight studies (Table 3). One woman described her ‘*up and down relationship’* with her own mother and outlined: *‘My mom, she drives me up a wall but she’s my rock’*. [14]. Conversely, a source of stress for pregnant women was being told what to do by their family; *‘I think what stresses me out is when people try to tell you what you can and can’t do. You know ‘you don’t need to eat that’* [11]. Similarly, women discussed stress related to their family giving unsolicited advice, as outlined by the authors of one study: *‘they are told that they should embrace traditional philosophy while they are pregnant and during parenting’* [1].

### Theme 2: Women’s experiences of healthcare

The impact of women’s experiences of healthcare on perinatal anxiety and stress was identified in nine studies (Table 3).

#### Perceived poor care from healthcare professionals (HCPs)

Poor care from HCPs was discussed as a major contributor to anxiety and stress by pregnant women and mothers. Women consistently reported dissatisfaction with the level of HCP support provided as expressed by one woman following her birthing experience: *’I was hyperventilating and although I’d had oxygen mask in theatre... I was given no such support in my after care. I felt neglected and terrified’* [4]. Some women described HCP behaviours as ‘*offensive’* [1], *‘intimidating’* [1], *‘dismissive’* [19] and ‘insufficient’ [41]. Women’s existing worries were often exacerbated following interactions with their HCP [4, 26]. For example, one woman felt anxious following insensitive treatment by a HCP: *“He then went to say: ‘I have two big problems with you – your age and the fact it’s an IVF pregnancy … the way he said it was awful”* [4]. Women also reported feeling anxious when HCPs would not provide them with adequate information, or they felt information was being withheld [24, 26]: *‘It’s almost like they treat you a bit like a child... like you can’t hear anything scary because you won’t be able to cope with it’* [24]. While women’s experience of care from HCPs was largely negative, women reported positive relationships with their HCPs in one study using words such as *‘supportive’* and ‘*helpful*’ to describe their nurses [26].

#### Access to healthcare services

In four studies, women expressed frustration and dissatisfaction with the quality of healthcare services available during pregnancy and postpartum (Table 3). One mother expressed dissatisfaction with the healthcare services available in public hospitals: *‘In my previous delivery, I went to a public hospital and tolerated bad circumstances... I don’t want my previous experience be repeated’* [2]. Similarly, in one study, although women mentioned that they had access to a wide range of services during pregnancy, they felt the quality of services available was a major problem [26].

#### Childbirth experience

Women in four studies described the experience of childbirth as anxiety provoking (Table 3). For women, particularly those experiencing a first pregnancy, perceptions of childbirth were characterised by uncertainty and women felt they could not truly establish a sense of certainty or control until their baby was born [2, 3, 19]. One woman voiced her fears of arriving late to the hospital: *'I am permanently anxious and ask myself what will happen if I don’t arrive at hospital on time? What may occur if I arrive late and my amniotic sac ruptures*’ [2].

### Theme 3: Factors that impact on coping

This theme examines some of the factors and behaviours that may facilitate or hinder women’s coping during pregnancy and the postpartum period.

#### Behavioural strategies

Women used a range of behavioural strategies to cope with their anxiety and stress, including comfort eating and talking. In one study, women discussed engaging in comfort eating when stressed; *’I eat more when my kids are stressing me out. I go straight to the kitchen’* [11]. Conversely, women in four studies found talking about their anxiety and stress helpful [3, 7, 11, 19]. One woman spoke about the importance of talking to effectively manage her stress: ‘*I have to talk about it. If I don’t say nothin’ about it, I’m just going to let it all build up ... as long as you have someone to talk to, it’s not as hard to cope with’* [7]. Some women struggled to talk about their anxiety and stress, which often led to feelings of loneliness and isolation. As the authors of one study explained: ‘*They keep their personal stress to themselves and feel they have no one to tell who will understand their dilemmas’* [1].

#### Faith

Pregnant women in three studies relied on faith as a method of coping with their anxiety and stress (Table 3); ‘*All I do is to separate myself from everyone and offer my prayers to God. This makes me stay relaxed and calm’* [3]. One woman, anxious because she felt her stomach was not growing enough to have a healthy baby, discussed turning to prayers as a method of coping: *‘Honestly, my other help was from prayers only. When I pray I get peace of mind. I stopped worrying’* [26].

#### Information

In two studies (Table 3), women reported anxiety and stress arising from the conflicting, confusing and inconsistent information they had been exposed to throughout pregnancy and the postpartum period. Mothers reported feeling anxious when their children did not conform to guidelines; *‘(milestones) put pressure on you … why is my baby not doing this? And then you start to Google if he doesn’t sit by this month what’s wrong with him?’* [24]. Receiving conflicting advice also made women feel they did not know who to trust; ‘*The midwife said one thing, the doctor said another, the two antenatal classes (I went to) gave exact opposite advice... I don’t know who to listen to’* [24]. One woman expressed the need for more readily available information to help manage her anxiety: ‘*Just like more information about what happens after you have the baby … like a little factsheet because I think that’s why you get the anxiety isn’t it’* [24].

### Theme 4: Social Norms and Expectations

In 10 studies (Table 3), women discussed anxiety and stress due to feeling pressure to adhere to perceived social expectations and ideas about pregnancy and early motherhood.

#### Being a ‘good mother’

In four studies (Table 3), women spoke about feeling judged, particularly in terms of their ability to be a *‘good mother*,*’* if their experiences did not fit with perceived social expectations. Women discussed feeling expected to embody unrealistic ideals of motherhood that did not recognise the realities of pregnancy and motherhood. For example, *‘I feel... like a failure … the fact that I could not breast feed raised the question in my mind about my ability to be a mother’* [41]. Stress related to the concept of being a *‘good mother’* was also tied to women’s ability to financially support themselves and their children; *‘I just stress that I can’t give her what she needs … I worry a lot about money’.* [41]

#### Mental health stigma

Norms and the stigma associated with perinatal mental illnesses were a source of anxiety and stress, and a barrier to seeking help in four studies (Table 3). Women felt embarrassed about their mental health difficulties, often hiding their symptoms due to the fear that they would be perceived as a *‘bad mother’* [24]. For example: *‘ … being a “good mother” is not compatible with mental illness; and having anxiety must make you a “bad mother”* [24]. One woman described social pressures to feel and act a certain way; *‘ … about your friends giving the perception that everything’s wonderful, you almost feel like you have to be’* [19].

#### Role changes and responsibilities

Adjustment to and assimilation of a motherhood identity was a source of anxiety and stress for women in nine studies (Table 3). Women in three studies felt stressed about adjusting to life with a baby and felt over-burdened by roles and responsibilities [1, 3, 4]. Mothers experienced stress juggling responsibilities with a new baby, and many verbalized struggles and difficulties with time management; for example: ‘*I feel I am neglecting him [older child] while I am dealing with her [baby] … the overriding factor is guilt’* [4]. Other women described the role of being a mother as *‘rough’* [4], *‘difficult but rewarding’* [14],*’frustrating’* [26] and *‘overwhelming’* [3]. Women also expressed lack of confidence in their abilities to be a mother; many felt out of their depth and uncertain about their choices and actions [43].

### Theme 5: Women’s and Baby’s health

The health of both woman and baby was discussed in six studies (Table 3).

#### Women’s health

Women in three studies experienced health problems in pregnancy and the early postpartum period that led to anxiety and/or distress (Table 3). For instance: *‘I felt bad and I was ill. About my health, after seeing myself very thin since... though it (was) for all my pregnancies but in this one it was severe with a lot of stress’* [26]). Women experienced anxiety because they faced difficulties taking care of their own needs, i.e. self-care [14, 26, 46]. One mother expressed that she did not *‘have time to take care of myself’* [14]. Additionally, many women expressed anxiety over making sure their body was as healthy as possible*; ‘Appropriate weight gain: too much, too little, when it happens, etc., eating the right foods, getting the right nutrition’* [46].

#### Baby’s Health

Women in four studies felt anxious and stressed about their child’s health in-utero and after childbirth (Table 3). For instance, concerns over the health of their unborn baby included; ‘*...an underlying fear that something will happen to the baby . . . I don’t think I’ll relax till he/she is here’* [46]. Older maternal age was also a factor affecting women’s anxiety about the health of their unborn baby; *‘My age is a big factor in the odds of having a baby with a genetic disorder’* [46]. Women also perceived infant health problems as a major source of stress and discussed struggling to cope with their baby’s health problems, leading to feelings of despair and helplessness. For example, ‘*My eight week old daughter has colic... for hours she squeals on and off. I find it hard not to get irritable … I know this is not her fault and that she needs comfort but I feel useless’* [4].

## Discussion

Unrealistic social norms and expectations, social support, poor healthcare experiences and concerns about health were identified as influencing experiences of perinatal anxiety and stress. Based on the GRADE CERQUAL approach, assessment of confidence in our findings is moderate to high due to the high volume of good quality, coherent studies relevant to this review question.

In line with previous research (e.g [28]), this review identified that socially constructed ideas of motherhood and unrealistic expectations of pregnancy and motherhood can lead to anxiety and stress when women’s experience does not meet their expectations [12]. This review also found that most mothers reported concerns related to adjusting to the role of becoming a mother, either for the first time or in the context of multiple children. Across studies, women felt overwhelmed by challenges of changing roles and responsibilities, and difficulties of, balancing competing demands of motherhood and self-care. The finding of a perceived overabundance of information available could increase anxiety by shifting the focus to women’s responsibility to educate themselves to get it right [25]. Lack of access to realistic and unbiased information about pregnancy and motherhood also resulted in differences between expectations and reality which, as discussed, contributes to perinatal anxiety and stress. Prioritisation of women’s mental health, including informing women that is it not unusual to feel overwhelmed is therefore critical.

Social support during the perinatal period was, unsurprisingly, identified as important for influencing women’s feelings of anxiety and stress. In this review, women felt dissatisfied with the level of support from their significant other which is consistent with previous work [27]. Male partners also experience distress during the perinatal period, potentially impacting their ability to support their partner [15]. Supporting paternal perinatal mental health therefore has benefits for men and provides opportunities to maximise effective support for mothers [15]. A strong desire to talk and engage with peers about aspects of pregnancy and motherhood to help cope with feelings of anxiety and stress was also identified in this review. This is in line with suggestions that observing that other women experience similar feelings is critical to reducing stigma associated with perinatal mental illnesses [55]. Moreover, our findings revealed that the family was both a source of support and a major source of stress for women, confirming previous findings [40]. Generally, women felt less supported in the postpartum period than during pregnancy in terms of health care support. This reflects previous findings that inattention, poor care from HCPs and inadequate hospital facilities are particularly problematic [6, 9]. Review findings also indicated that women’s negative experiences of the healthcare system were related to poor perceived quality of services available, suggesting a need for greater investment in resources available in antenatal and postnatal care. Increasing investment in antenatal and postnatal resources is difficult to achieve however [10].

In addition to interpersonal and structural supports, supporting women to engage in effective coping strategies is important for perinatal anxiety and stress [18]. Similarly, to previous research ([32] Thomas et al), engaging with faith and/or talking to others were identified as strategies women already use to reduce anxiety and stress. The finding that some women comfort eat to cope with perinatal stress may reflect a negative coping mechanism because overconsumption of food during pregnancy increases the risk of excessive weight gain and gestational diabetes [52].Women’s perceptions of their infants as susceptible to compromised health was also identified as a source of anxiety and stress in this review, which is in line with previous findings highlighting associations between fear of the unknown during pregnancy and labour and anxiety and stress [23, 36]. Women also reported a range of post-childbirth complications and described anxiety and stress related to their health concerns. This is important given that potential bidirection relationships between perinatal mental health and health status have received little research, policy, and clinical attention [49]. Greater consideration of maternal and infant health impacts on maternal perinatal mental health in future research and practice is therefore needed.

### Strengths and Limitations

This review used a comprehensive literature search strategy to maximise the identification of relevant articles and used of the GRADE CERQual to provide overall levels of confidence for each of the review findings. However nearly all included studies were conducted in high income countries and cultural differences in experiences of anxiety and stress may not therefore have been captured despite the inclusion of papers in any cultural setting [22]. Despite the predominant focus on developed countries, the primary studies in this review included diverse ethnic and socio-economic groups, enhancing the generalisability of our findings.

## Conclusion

This review highlights that women experience perinatal anxiety and stress due to inadequate social support, poor healthcare experiences, unrealistic social norms and expectations, and health related concerns. There is a need for greater focus on perinatal anxiety and stress in research and practice. At a structural level, supporting HCPs to support women’s mental health (i.e. through appropriate training) is essential. At the societal level, addressing socially constructed ideas of motherhood that contribute to unrealistic expectations, is an important step towards better supporting women. Finally, enhancing women’s social support networks and provision of clear, consistent information are essential to support women and minimise anxiety and stress in the perinatal period.

## Supplementary Information

**Additional file 1:.**

**Additional file 2:.**

**Additional file 3:.**

## Data Availability

Not applicable. However, the data that supports our findings can be found in the additional supporting files (supplementary materials)
