# Supplementary material for: Women’s experiences and perceptions of anxiety and stress during the perinatal period: a systematic review and qualitative evidence synthesis
Source: BMC Pregnancy Childbirth. 2021 Dec 6;21:811. doi: 10.1186/s12884-021-04271-w (PMC8647378; doi:10.1186/s12884-021-04271-w)
Supplement: Supplementary file 1 — Additional file 1:. [file 12884_2021_4271_MOESM1_ESM.docx]

| **Study Information** | **1** |
| --- | --- |
| Title | Women’s views on anxiety in pregnancy and the use of anxiety instruments: a qualitative study |
| Publication year | 2017 |
| Author | Evans et al. |
| **Participant Characteristics** |  |
| Participant group: Pregnant women/mothers (of children up to one year) | Women who have given birth within 9 months postpartum |
| Number of children | not given |
| Age | not given |
| Ethnicity | not given |
| SES | not given |
| Recruitment strategy | Local healthcare professionals (HCPs) were contacted and provided with details of the study. The HCPs were asked to introduce the researcher to eligible women and obtain permission for the researcher to make contact to provide further information. Once permission was received, women were sent written information outlining the study and had an opportunity to consider their involvement and ask any questions. |
| Sample size | Two groups of women participated in the study. Focus group one included 15 women and focus group two included 4 women. |
| Eligibility | To be included in the study, women had to be at least 18 years of age and have given birth within the previous nine months to a healthy, full-term infant. Women were excluded from the study if they had received treatment for a severe and enduring mental health condition; did not speak, understand, read and write in the English language; or were pregnant |
|  |  |
| **Methods** |  |
| Study aim | The aim of the study was to explore women’s experience of anxiety in pregnancy and their views on the use of anxiety instruments in antenatal care. |
| Theoretical background | not given |
| Design | A qualitative methodology using focus group discussions (FGDs) was employed |
| Study setting | Community setting and hospital clinic setting |
| Data collection approach | Focus group discussions |
| Interview/focus group duration | One and a half hours |
| Use of additional resources/prompts | No |
| Analysis | Template analysis was employed to enable an investigation of the data to identify new concepts but also to interrogate the data for themes identified in the literature. |
| **Findings** |  |
| Theme | **Theme one: sources of support** *Women were not able to share their feelings of anxiety with others and felt isolated-* Barriers to disclosing their feelings included not wanting to burden or cause concern to family members. Although supportive, husbands and partners found it difficult to sympathise with their situations. When asked who they talked to about feeling anxious, no participants in focus group one mentioned their midwife. Some participants identified a lack of continuity of carer and midwives’ busy schedules as reasons they had not been able to develop a good relationship with their community midwife. Women in focus group two had tried to disclose their anxieties but felt that their midwife did not understand or tried to minimise their feelings. Difficulty in discussing feelings resulted in women from both groups expressing a sense of isolation. Focus group two participants described how they had often struggled with identifying why they were anxious. They knew other women who had been through worse situations and this left them feeling foolish and embarrassed about their own feelings. Women from focus group one found the pressure to conform to the image of a happy, healthy pregnancy led them to feel they were abnormal and made them more reluctant to share their true feelings. *Women felt that connecting with others with similar feelings would help-* Many participants would have liked to talk to others who were going through similar experiences; peer support would have encouraged them to share mutual experiences and therefore feel less isolated. Having someone who could empathise with them would have helped them to accept it was normal to feel the way they did, and that other people understood those feelings. *Women received additional support at a time of crisis-* Women in focus group two who had access to support for anxiety in pregnancy, described how they found it impossible to hide their feelings. Some had tried to discuss their feelings ‘... and that can be worse if somebody tells you... oh yeah don’t worry about it it’s almost like they’re wishing it away. Sometimes although you might be worrying for no good reason it’s just nice for someone to say yeah I understand’ [G1/P6] ‘answering a generic questionnaire really doesn’t feel right to me because I feel anxious because I’m pregnant... It reflects my anxieties and worries the best. I don’t feel anything about the others; I like this one the best... I would have loved to have had this questionnaire’ [G1/P2] ‘it’s good to break it down into different feelings... sometimes it’s hard to explain or because you’re in a bit of a state ... [referring to the HADS instrument] it’s like oh Yep, yep, yep, all of those’ [G2/P3] ‘... with the questionnaire perhaps people who are worried about the same things maybe give you a connection so you can meet up and talk about it... say well I feel the same as you, so rather than you feeling isolated you’ve a got someone to talk about it to, and maybe have someone there ... a professional who would balance the worries out’ [G1/P4]. ‘You have to be an extreme, there’s not much for the people in the middle. Like this time there wasn’t really that much other than (specialist midwife). It was the same last time. Once I’d got past wanting to kill myself, once that had gone, there wasn’t really anything much for me’ [G2/P4] . |
|  |  |
|  |  |
|  |  |
|  | **Theme three:** Instruments prompting discussion**-**  *Relevance and wording of questions:* When presented with examples of anxiety instruments, participants thought some items could be beneficial in helping women express their feelings, although other items appeared to minimise their feelings or were considered irrelevant in pregnancy. Some EPDS questions (Cox et al., 1987) have a response option of ‘for no good reason’ (e.g., I have been anxious or worried for no good reason). Participants did not like this wording as they felt they had good reasons for their fears and anxieties. For some women in focus group one the PRAQ pregnancy-specific instrument (Van den Bergh, 1990) was considered helpful to women experiencing severe symptoms but did not reflect their own feelings. They thought women who identified with the PRAQ statements must be ‘really struggling’ [G1/P5], although they acknowledged that statements such as ‘I am afraid the baby will be stillborn or die’ could help women feeling these emotions to express their fears honestly. A number of participants from focus group one and all focus group two participants could identify with the statements and suggested that it reflected anxieties specific to pregnancy.  *Helping to clarify and discuss anxious feelings:* Participants in group two identified with the concepts in the HADS (Zigmond & Snaith, 1983) and liked the way the sentences were presented (e.g., ‘Worrying thoughts constantly go through my mind’) in that it separated their anxiety into individual topics. They said it was easier to talk about their feelings if they could identify with the statements. Reading the individual statements in the anxiety instruments prompted discussions in both groups about the nature of anxious feelings. *Helping to identify what would help:* The discussion of anxiety instruments led to participants identifying aspects of additional support which may have been welcomed. Women in focus group one found their ongoing postnatal group to be a good source of support and recognised that peer group support would have helped them feel less isolated and would have provided reassurance during pregnancy. They suggested that midwives could facilitate group meetings with women who were experiencing similar feelings. Women from focus group two also placed importance on meeting others in similar situations, although this would have been preferred alongside individual support. They felt that some pregnant women may have been willing to self-refer to additional services if information about access had been available. However, women considered that the amount of support for women experiencing mild or moderate symptoms of anxiety was limited. |
| Discussion | Women often lacked the professional support they needed to discuss their anxieties and would have welcomed a greater focus on their emotional health in pregnancy. They attributed inadequacy of emotional support to constraints on midwives’ time due to heavy workloads, a lack of awareness of supportive services and problems with continuity of care. Previous studies have reported that midwives have similar concerns which they identified as barriers to providing effective care for pregnant women with mental health problems (Jarrett, Green, & Spiby, 2009). To facilitate an effective discussion of women’s emotional health, midwives need sufficient time to build a relationship with the woman to enable her to discuss her feelings and access professional support. Women in this study considered that the use of anxiety instruments would have helped to prompt an open discussion with their midwife. Improving midwives’ awareness and knowledge through training and an understanding of referral processes and support services can empower midwives to support women with perinatal mental health problems (Jomeen et al., 2012). The women were reluctant to share their anxieties with family members, to avoid causing emotional burden. They felt they had to conform to having a positive pregnancy experience. Participants identified additional support that would have been helpful to them. Other studies have described how pregnant women often turn to informal support to meet their emotional needs (Raymond et al., 2014). Women in both groups concluded that peer support would have allowed them to talk openly without burdening or upsetting family members and would have confirmed that they were not unique in their experience. Observing that 86 K. EVANS ET AL. others have similar feelings has been described as a key component of group therapy which reduces stigma around anxiety (Whitfield, 2010; Yalom, 1995). Both groups discussed the use of anxiety instruments; the most positive comments related to the pregnancy-specific questionnaire (PRAQ-R, Van den Bergh, 1990). Participants confirmed that some of the questions surrounding the health of the baby reflected their emotions at the time. During pregnancy the women’s difficulty discussing their feelings meant they welcomed the anxiety questionnaires which presented the items in the first person and present tense, ‘I feel ..., I get ... I am afraid ...’. Women’s concerns about the use of anxiety instruments in pregnancy related to three areas: the information being used to highlight child protection concerns; completion of anxiety instruments as a tick-box exercise without realistic support and referral options; a positive professional relationship would be required to enable the women to provide honest answers. Clinicians should be aware that women’s worries and discomfort in disclosing details could affect the utility of anxiety instruments (Côté-Arsenault & Donato, 2011). Providing a supportive context for the administration of anxiety instruments may help women disclose their true feelings and seek support without concern about being misunderstood (CôtéArsenault & Donato, 2011). In the UK, it is a midwife’s duty of care to report concerns if they believe someone may be at risk of harm to themselves or others (Nursing & Midwifery Council, 2015). However, it should be made clear that the primary aim of instruments is to highlight when women may benefit from further support. It is important that midwives are fully aware of the implications of administering anxiety instruments in pregnancy. This includes providing women with the opportunity to discuss any concerns and signposting to appropriate support. Darwin, McGowan, and Edozien (2013) identified that psychosocial assessment during pregnancy helped to raise women’s own awareness of their emotional distress, encouraged self-management strategies and provided reassurance from having their experiences validated. In line with the findings of the present study, there were no positive benefits from psychosocial assessment when women perceived the assessment as having little importance to the assessor |
| Conclusions | The introduction of routine psychosocial assessment including the use of anxiety instruments may offer an opportunity to prompt a discussion around women’s emotional health in pregnancy and facilitate the offer of support to women who may benefit. All three instruments explored in this study were acceptable, but women preferred the style of questions and concepts in the pregnancy-specific instrument. This research found that women would welcome an opportunity to discuss their anxieties with their midwife and with other women with similar concerns. To create the right context for discussion, women must feel understood and midwives must have enough time to discuss any issues revealed. Midwives may benefit from further training to highlight the importance of discussing emotional health in pregnancy, acknowledge the difficulty women may have when expressing their concerns and develop the skills to address them sensitively. Pre-registration midwifery education programmes in England are now required to include a perinatal mental health module, with further provision for post-registration training |
| Limitations | A potential limitation was the size of focus group two. However, the benefit was that the small group size enabled the women to feel safe in discussing potentially sensitive topics with strangers |

| 2. Title | “It’s Hard Being a Mama”: Validation of the Maternal Distress Concept in Becoming a Mother |
| --- | --- |
| Publication year | 2019 |
| Author | Copeland et al. |
| **Participant Characteristics** |  |
| Participant group: Pregnant women/mothers (of children up to one year) | First time mothers who birthed a full-term infant aged between 6 weeks and 6 months |
| Number of children | not given |
| Age | Mean age of 21 |
| Ethnicity | Caucasian (95%) African American (5%) |
| SES | Low-income mothers |
| Recruitment strategy | A convenience sample of 21 low-income mothers were recruited from a Women, Infants, and Children (WIC) clinic |
| Sample size | 21 low-income mothers |
| Eligibility | First-time mothers were eligible to participate if they were 18 years of age, birthed a full term infant aged 6 weeks and 6 months with no post birth complications, and were able to read and write in English |
|  |  |
| **Methods** |  |
| Study aim | This study aimed to determine how maternal distress influences mothers’ transition to becoming a mother and to validate the use of the Maternal Distress Concept in the clinical setting |
| Theoretical background | Social Cognitive theory |
| Design | This qualitative study was a secondary analysis of a mixed-method, cross-sectional, study on maternal competence, self-esteem, and sense of mastery among first-time, low-income mothers |
| Study setting | Women, Infants, and Children (WIC) clinic that is located in a suburban area in southeast Louisiana |
| Data collection approach | Semi structured interviews |
| Interview topics/focus | Women’s experiences |
| interview/focus group duration | 15-30 minutes |
| Use of additional resources/prompts |  |
| Analysis | Directed content analysis was used to analyse interview data |
| **Findings** |  |
| Theme | **Stress Attribute** *Emotional Concerns:* Mothers reported a variety of emotions when describing their experience with the infant while at home and used certain describers such as “stressful,” “scary,” “overwhelmed,” “crazy,” and “difficult” to describe this experience. One mother said “it was scary home alone on first day but exciting to wake up and see her” (mother #1). However, another mother said she “gets upset sometimes over little things and am still nervous” (mother #2) while another mother reported the following issue demonstrating low mood: “I know when I first came home the postpartum hit me a little but it wasn’t anything horrible” (mother #12). One mother was stressed due to social and environmental changes in her life: “I’m a little stressed because we’re trying to buy a house and getting married in July [and] my fiancé and me are living with my mom, which can be frustrating” (mother #14). Another mother was stressed due to a feeding issue and explained, “The first 3 days I felt crazy. I didn’t know what she wanted. I was breastfeeding at first and didn’t know if she was getting enough and she was crying” (mother #3). Another mother stated she had bad dreams “that something was going to happen to him, like I burp him and he spit up or choke, or, I was going to wake up and he was not breathing—just scary new-mother stuff” (mother #5). *Pregnancy:* Even though the interviews were completed during the postpartum period, several mothers addressed their emotional state during pregnancy. One mother stated that she was scared during pregnancy and another mother stated she was very emotional during pregnancy. One woman stated, “Emotionally, yeah. I’m like, when I was pregnant it was bad, but I never got over that” (mother #18). Overall, no mothers reported they were diagnosed with depression in pregnancy or postpartum but one mother stated that “through my pregnancy, I was actually kind of depressed a lot and scared, but my views and feelings have changed so much” [meaning in the postpartum period] (mother #16). For some mothers, pregnancy was a time of emotional turmoil that still resonated with them in the early postpartum period. *Managing Life and Relationship***s:** The majority of mothers expressed concerns over how pregnancy will affect their lives and family relationships. For example, one younger mother stated: I was very scared. I was still in high school and I waited until after graduation to tell my parents that I was three months pregnant. I was really stressed at the beginning and was scared that her dad [the baby’s dad] wasn’t going to stick around. We’d only been together for a year. But he surprised me because he works every day and he supports her. (mother #14) Another mother stated “I guess the hardest thing is trying to manage everything, doing a bunch of things, as well as taking care of her [the baby]. Cause I’m still in school. I’m just trying to manage my time and stuff now” (mother #20). In addition, one mother verbalized frustration with incorporating the baby into her family business work schedule. She states, “So, I’m with the baby 24/7 and I bring the baby to work but can’t get any work done” (mother #9). Becoming a mother involves transition and change and as one mother summed up, mothers think about how the baby will change their lives. She states: When I was pregnant, I thought oh my God, my life is over. I’m never going to be able to do anything. I’m not going to be able to finish school. But that’s all changed. I figured out that everything is accomplishable. (mother #20) |
|  |  |
|  |  |
| Conclusions | The process of becoming a mother is usually perceived as a happy event, one that requires many changes and adaptations to successfully attain the maternal role; however, as this study and others (Barclay et al., 1997; Barclay & Lloyd, 1996; Emmanuel & St John, 2010; Fenwick et al., 2013) indicate, many mothers may find this process quite distressful, depending on the adequacy of resources and support. In the perinatal period, it is important to view emotional distress as more than a psychiatric diagnosis of depression, anxiety, or another psychiatric disorder, but to recognize that new mothers experience dramatic lifestyle changes and adaptations when becoming a mother that include incorporating an infant into her family and social system. Further, HCPs must inform new mothers that it is “normal” to have some level of distress when becoming a mother so the mother will be more apt to disclose her feelings and receive help and resources from her social support and professional networks. For HCPs, the Maternal Distress Concept analysis is very useful for assessing mothers’ distress levels in the domains of stress, adapting, functioning and control, and connecting as she transitions to becoming a mother. The results of this study confirm the work of Emmanuel and St John by providing an alternate view of maternal distress and serve as a building block to a descriptive theory |
|  | New mothers’ perceptions of maternal distress in early parenthood varied on a continuum and were influenced by a variety of factors. Mothers reported concerns related to stressful events, adapting to the maternal role, recovering from childbirth and taking care of self, and connecting with social support networks. Overall, most mothers reported some level of maternal distress, but the group as a whole expressed lower levels of maternal distress. Even these lower levels of distress required mothers to adapt to the mother role, as demonstrated by their interview responses. Becoming a mother is often a nonlinear path and mothers will experience various degrees of maternal distress involving changing social circumstances, adaption to the maternal role, recovery from birth, and maintaining and/or reintegration of partner, family, and social networks. No mothers in the interviews stated that they were diagnosed with a mood disorder in pregnancy or in early parenthood, but this reality cannot be validated since a question on mood disorders was not asked in the original study. However, the findings in this study revealed that most mothers reported some type of concern related to becoming and adjusting to the mother role. Further, the findings reveal a new perspective on maternal distress that is not related to psychiatric diagnoses, but rather “normal” feelings and adjustments to becoming a mother that affect mothers’ emotional well-being. In this study, mothers’ perceptions of stress related to their emotional concerns with being a new mother, managing change in their social and environmental events, and verbalizing distress in pregnancy were noted. These feelings are supported by other researchers that assert that mothering elicits changes in emotional well-being associated with the transition to the maternal role. Although maternal attitudes in pregnancy can be related to severe mood disorders, such as depression and anxiety, in the postpartum period,it is important to assess the total experience of becoming of a mother, rather than only mood disturbances. It is important for HCPs to encourage mothers to talk about their pregnancy and birth stories in the postpartum period so mothers can obtain a better understanding of their fears, concerns, unclear understandings about pregnancy and birth events, and feelings of inadequacies or disappointments. Further, the concept of maternal distress should not be strictly assessed as a medical problem, but rather as a normal response to becoming a mother (Arditti et al., 2013). It is expected that mothers will experience various levels of maternal distress in becoming a mother due to a variety of factors. |
| Limitations | The limitations of this study include use of a homogenous sample, secondary analysis of previous data, and cross-sectional design. The sample was predominately White and the majority of mothers were single but this sample described the usual race of a WIC population in which 58.7% are White, as reported in the WIC Participant and Program Characteristics Final Report. Also, secondary analysis of previous data was used and richer results may have been obtained if all interview questions were directed at obtaining information on maternal distress. Finally, the initial study data were collected using a cross-sectional design with no opportunity for follow-up questions. |
| Recommendations | Not given |

| 3. Title | Supporting perinatal anxiety in the digital age; a qualitative exploration of stressors and support strategies |
| --- | --- |
| Publication year | 2020 |
| Author | Harrison et al. |
| **Participant Characteristics** |  |
| Participant group: Pregnant women/mothers (of children up to one year) | Women who were either pregnant or within one-year postpartum |
| Number of children | not given |
| Age | 20-45 years |
| Ethnicity | Mixed Ethnicities |
| SES | not given |
| Recruitment strategy | Convenience sampling was used to recruit participants through advertising on social media and relevant forums (e.g. MumsNet), and via local mum and baby groups |
| Sample size | 23 women who were either pregnant or within one- year postpartum |
| Eligibility | Inclusion criteria were that women [1] were pregnant or had had a baby in the last 12 months; [2] were aged 20– 45 years; [3] lived in the UK, [4] were fluent in English; and [5] had experienced anxiety during the perinatal period |
|  |  |
| **Methods** |  |
| Study aim | The aim of the current study is to use a qualitative approach to explore women’s experience of PNA, in particular, considering the main sources of their anxiety (i.e. triggers) and the support/coping strategies they use in both online and offline contexts. the aim of this study is twofold. First, to qualitatively explore women’s experience of anxiety triggers and support in the perinatal period; and second to gain insight into what online support is acceptable for women with PNA. |
| Theoretical background | not given |
| Design | A qualitative study using focus groups |
| Study setting | community centres in different geographical locations around the UK |
| Definition/conceptualisation of stress and/or anxiety | not given |
| Data collection approach | focus groups |
| Analysis | Data were analysed using inductive thematic analysis from a realist stance |
| **Findings** |  |
| Theme | **Expectations of breastfeeding** A primary source of anxiety (and a topic that spontaneously arose in all FGs) was how unprepared women felt for the realities of breastfeeding. Many reported being “surprised”, “shocked” and “anxious” when they found breastfeeding to be “one of the hardest things I ever had to do.” (FG1.F5) and felt this was a direct result of the mismatch between their struggle in reality, and the information presented by antenatal classes, HCPs, social and mainstream media, which led them to expect breastfeeding would “be a magical experience”, “come naturally” or “be easy”. Overall, women felt let down by the biased information they received about childbirth and breastfeeding and expressed a need for more balanced and realistic information. FG5.F4: Why is this kind of bias allowed in a place where you know, you’re actually working with people who are quite vulnerable… people should be able to provide unbiased information about childbirth and breastfeeding. |
|  |  |
|  |  |
|  |  |
|  | **Unrealistic guidelines and norms** A further source of anxiety came from unrealistic guidelines and norms, for example the developmental milestones frequently reported on mum-focused websites (such as Bounty, BabyCentre etc). These resources aim to give parents a guide of what their child ‘should’ be doing at different ages. However, many of the mothers reported experiencing anxiety when their children did not show all of the skills on these lists. FG1.F5: (milestones) put pressure on you. Because you want to know, ok…why is my baby not doing this? … And then you start to Google if he doesn’t sit by this month what’s wrong with him? Many women also felt childcare guidelines were often “unrealistic” (particularly on the topics of sleep and feeding) and offered no alternative suggestions for situations when their babies did not adhere to the guidance advice |
|  | **Unrealistic social comparison** Social media was a particular source of anxiety, as it promoted unrealistic expectations of motherhood through social comparison. When faced with images of other mothers (either friends, family, celebrities or strangers) seemingly able to ‘bounce back’ immediately after childbirth, effortlessly managing to balance motherhood with other aspects of their life, and do everything ‘right’, many mothers reported experiencing intense feelings of failure and worry about their ability as a mother |
|  | **Importance of peer support**- **Offline social support** Most women spoke about motherhood being isolating at times, which was a large source of distress. Having a supportive family and partner was described as being important and stress relieving, whilst an absence of support was often experienced as distressing. Participants consistently highlighted their peers as the single most important source of support. In most cases this was explicitly discussed in terms of face-to-face social support groups, such as antenatal classes (e.g., national childbirth trust, baby and bump, etc.) or breastfeeding cafes. FG5.F3: I would say this (antenatal group) has been the most important thing terms of support really. However, many of the FG participants did not take part in antenatal classes, primarily “because it’s so expensive...” (FG3.F1), which often led to feelings of isolation. Interestingly, this arose as an issue more in the rural FGs than the urban groups and may be indicative of social-economic differences between the groups. In contrast to antenatal groups and breastfeeding groups, postnatal ‘mum and baby’ classes were not identified as an important source of support and were often experienced as isolating, as they do not encourage adult peer interaction (due to their primary focus on the babies). FG2.F1: I’d go to these classes… (but) people wouldn’t go by themselves, they’d all go in twos. And I’d sort of sit there and I’d be going I haven’t spoken to anyone. Furthermore, discussions suggested there were fewer mum-and-baby classes available in rural (compared to urban) settings. (FG1.F5: if you live maybe in a small village, then I think you don’t have that much possibility to have these baby events.) And some mothers reported being “denied access to postnatal groups” (FG2.F2) because they already had older children. |
|  | **Online social support** Opinions of online support networks were somewhat mixed. Generally speaking, large scale forums were predominantly seen as anxiety-provoking, rather than relieving; while smaller online support groups (for example, Facebook groups with a single support focus, such as breastfeeding, pumping or gestational diabetes) were often characterised as being helpful. Participants often explicitly referred to this type of peer support as being more helpful than other, more professional, avenues of support. FG3.F2: Mumsnet gives me a little bit of anger… FG3.F1: …Yeah, sometimes it makes you worry more. FG2.F1: I would use (the Facebook group) for support there because I found, I tried the midwife, I tried the health visitor, I tried family and I tried the GP, and I didn’t really get anywhere. So, for me I used that because I don’t really… leave the house all that often...But actually I think the biggest support and the biggest help people get is other mums that are going through exactly the same thing |
|  | **Maternal confidence and overwhelm** In all FGs women alluded to an initial uncertainty about their maternal competence, with many suggesting that they felt out of their depth, and uncertain about their choices, feelings and behaviours. FG1.F6: People always just say just trust your instincts, you’re the mother you know best. But I don’t know. I don’t have any instincts because I don’t know about this situation. So, I actually find that… to be really anxiety inducing. Women often described feeling “overwhelmed” (FG3.F2) and found it difficult to balance the demands of motherhood with those of everyday life. Conversations often fell into ‘before/after’ childbirth narratives, with mothers often feeling that they were no longer able to do the things they used to, including finding the time to look after themselves. FG3.F1: I never prioritise (looking after myself) anymore but I should… I just can’t stop enough… to do it. |
|  | **Conflicting (or extreme) information** Online sources of parenting information were often described as anxiety-provoking. The overabundance of information and polarising opinions online left women feeling confused, and not knowing who to trust (see Table 2). Confusing, conflicting information was also experienced in an offline context, with women frequently reporting receiving different guidance from HCPs. Mothers felt that these were usually just “people’s subjective opinions” and felt there wasn’t enough evidence based, unbiased and/or middle-ground information to address their concerns. FG5.F3 “The midwife said one thing, the doctor said another, the two antenatal classes (I went to) gave exact opposite advice, and don’t even get me started on the internet. That just seemed to be one extreme or the other… So, I was like, I don’t know what to do…. I don’t know who to listen to.” Interestingly, while women felt under pressure to live up to socially constructed ideals of motherhood, the presence of conflicting advice reveals how there is not really one true ideal. Not only is this confusing (and inherently contradictory), but also has the propensity to position women as always being wrong, regardless of what they do and what advice they follow |
|  | **Internal and external** **stigma-** Several women reported feeling ashamed or “embarrassed” of their PNA symptoms, demonstrating internalised stigmatising attitudes directed towards themselves. This was usually accompanied by a demonstration of external stigma (i.e., having concerns about how others would see them if they told them about their anxiety). For example, many women spoke about deliberately hiding their symptoms from others, as they were worried that they would negatively judge them and/or their parenting abilities if they found out about their symptoms |
|  | **Disclosure avoidance** Disclosure avoidance related to women’s concerns about seeking professional help. Related to internal and external stigma, many women felt reluctant to disclose their symptoms to HCPs. This was largely due to a fear that they would be seen as a bad mother, and that there may be significant negative consequences as a result (for example, worrying that their baby may be taken away from them, or that social services might intervene). Thus, stigma acted as both a source of anxiety and as a barrier to help-seeking behaviours. Apparent in both categories of stigma is a dichotomy that implies the notion that being a “good mother” is not compatible with mental illness; and having anxiety must therefore make you a “bad mother”. |
|  | **Mental health literacy** Lack of knowledge about PNA and about maternal mental health was also an anxiety-inducing factor, and again acted as a barrier to seeking (or receiving) support. Many women reported feeling distressed about being unable to find any information about the way they were feeling either on- or off-line. In many cases they explicitly reported that they did not think that they had PND, but that they “didn’t really know the difference between PND and anxiety” (FG2.F1). These women said that they did not identify with PND symptoms but were unable to find an alternative explanation of their symptoms which made them feel like they were “just going mad” (FG5.F3); “I didn’t know what was wrong. I thought I’d just lost the plot.” FG3.F2. Several mothers reported feeling a significant sense of relief once they were able to identify that they probably had PNA (either via HCP intervention or self-diagnosis), highlighting the role of uncertainty in anxiety: FG5.F1: Once I knew that was happening it was easier… because I knew what to expect. And many mentioned that knowing what was wrong acted as a facilitator for access to support. However, even when it was recognised, some women felt there was a lack of information about how to cope with PNA, and what they could do to relieve symptoms. FG2.F1: For me I just haven’t found… coping strategies. As in if you’re having an anxiety attack it’s quite hard to have that when you’re looking after children… What’s the best thing to do when you have an anxiety attack? Overall, several common sources of anxiety were identified by the participants, and a number of issues around current support were highlighted. |
|  | **Better preparation/management of expectations of birth and motherhood** Women found that much of their anxiety stemmed from a lack of maternal confidence, which was often related to the uncertainty they felt as a result of the often unrealistic, confusing and inconsistent information they had been exposed to about major aspects of motherhood. To help counter this, women frequently stated they wanted access to unbiased, balanced and realistic information about important aspects of motherhood, namely labour/childbirth, breastfeeding and sleep management. Where possible, they wanted to see the evidence-base behind claims and guidelines, so they could understand their origins and enable them to make more informed choices about their actions. While they wanted expert advice on these matters, they also wanted access to realistic perspectives in the form of moderated peer input (limited to avoid the replication of problems seen in big forums, such as the presentation of too many opinions, and extreme opposite views). |
| Discussion | This study qualitatively explored women’s experience of anxiety triggers and support preferences in the perinatal period. The broad aim of this study was to use a bottom-up qualitative approach to identify common sources of anxiety as potential targets for intervention and provide a useful framework for the development of acceptable online programs aimed at supporting PNA. To this end, the discussion will compare study findings to previous research, and synthesise the main themes, exploring current issues around support and discussing how to overcome them; and drawing together the main targets that were identified and the acceptable online solutions that may be able to address them |
| Limitations | Findings from this study may not be generalizable to all mothers who have experienced PNA. Women who took part in the focus groups were mostly white, educated, heterosexual, and in normative relationships with their child(ren) ‘s father |
| Conclusion | This work provides unique insight into potential sources of anxiety for women in the perinatal period, while also offering potential internet-based support solutions that are likely to be acceptable and helpful for women with PNA. |
| Recommendations | Further research should aim to include women from a wider range of socio-demographic backgrounds, relationship statuses, and internet usage patterns, and may want to consider using online interview methods to broaden the demographic reach by reducing the logistical constrains associated with attending face to face focus groups |

| 4. Title | Stressful events, social support and coping strategies of primiparous women during the postpartum period: a qualitative study |
| --- | --- |
| Publication year | 2009 |
| Author | Razurel et al. |
| **Participant Characteristics** |  |
| Participant group: Pregnant women/mothers (of children up to one year) | 60 women interviewed six weeks after the birth of their first child |
| Number of children | not given |
| Age | Mean age of 31 |
| Ethnicity | not given |
| SES | Mixed |
| Recruitment strategy | Mothers were recruited during their hospital stay (early post-partum) |
| Sample size | 60 women |
| Eligibility | All French-speaking women having their first child at more than 37 weeks of gestation after a normal pregnancy without pathology or hospitalisation were eligible for inclusion in the study. |
|  |  |
| **Methods** |  |
| Study aim | The aims of this study were to investigate events perceived as stressful by primiparous mothers during the postpartum period and perceived social support, and to identify coping strategies. |
| Theoretical background | not given |
| Design | A qualitative study design using semi- structured interviews |
| Study setting | Geneva University Hospitals, Geneva, Switzerland |
| Data collection approach | Semi- structured interviews |
| Interview topics/focus | Stressful events perceived by the women during pregnancy, birth and postpartum; perceived stress and perception of control of these events; received and perceived social support from family and friends and from maternity staff; coping responses of mothers during the postpartum period; and education received from health-care professionals |
| Interview/focus group duration | not given |
| Analysis | An iterative approach was used to construct an ‘analytical tree' |
| **Findings** |  |
| Theme | **Stressful events** Ten stressful events were identified in the early postpartum period and 12 in the late postpartum period. In the early postpartum period, the most stressful perceived problem was the interaction with caregivers, especially when the latter minimised the difficulties encountered by mothers and when they provided contradictory information. Breast feeding associated pain and feeding difficulties were considered as the second most stressful events. The third event concerned the conditions of hospitalisation, mostly related to the lack of rest and privacy. Most women expected to be able to rest during this time, but overcrowded conditions on the wards did not allow this. |
|  |  |
|  |  |
|  |  |
|  | **Breast feeding** At home, the major stressful event was breast feeding. Women’s perceptions were strongly negative, and pain, sometimes unbearable, was reported as a particularly important aspect of breast feeding: I could not see the end. I was completely physically and mentally exhausted. Each time he sucked the left side, I saw stars, I had a stress ball. I dug my nails into my husband’s arm. I had my husband beside me to tell me to breathe, because the pain was so bad that I stopped breathing, I held my breath and clenched my teeth, I was almost afraid to put the infant to my breast. (J03) There is a strong idealisation of breast feeding with important projections and stakes. It appears to be perceived as one of the indicators of a ‘good mother’: It was difficult to take the decision to stop breast feeding. Because I feel bad myself about not being able to produce enough milk and satisfy my baby. Yes, like a failure. I was not capable of. Then I said to myself, will I be able in general to take care of him? The fact that I could not breast feed raised the question in my mind about my own ability to take care of this child. Will our relationship be sufficient? All that, I linked to my breast feeding. (Ch1) Women considered breast feeding as a natural behaviour and focused on information given during prenatal education where the future difficulties of breast feeding are often concealed: Finally, it was mostly to do with breast feeding. No one told us about the disadvantages. It was only that it is very good, it is very satisfying. I had fissures on the second day. No one had told me about that. During the prenatal course, there were slides with attractive women breast feeding their child, but no negative aspects were mentioned, just that everything was going very well. Whereas me, I almost gave up. (M14) Most women perceived breast feeding as a negative event and were unable to achieve any degree of satisfaction. An additional major event concerned the practical organisation of breast feeding, and women reported this as a source of high anxiety with feelings of being completely overwhelmed. |
|  | **Social support during the early postpartum period** During the early postpartum period, women considered emotional social support, which includes help to maintain self-esteem, as the most important factor. ‘Interaction with the caregiver’ emerged as the major stressful event. Women were dissatisfied with the social support provided by health professionals and considered it to be either insufficient or unsuitable for their needs: The infant cried a lot. I called the midwife and she said ‘what do you want me to do?’ I was very disappointed. I needed someone to give me support. (M13) Similarly, emotional support was also perceived as the primary protective factor in relation to the second-most stressful event, breast feeding: When you encounter breast-feeding difficulties, you have the impression that nobody is listening to you, you are told to keep trying, everything will go well, and it is not important. It is a pity and it is very hard y. (M06) For the event ‘baby’s health’, informative support was considered to be the most satisfactory option if open-ended. Women want to be considered as partners in the care process, and if the informative support is directive or contradictory between professionals, it is perceived negatively. |
|  | **Social support during the later postpartum period** at home, the expected social support is diverse and of a different type. Faced with the practical organisational aspects, women feel great concern and express a tremendous need for material support. Although the privileged support base is the partner, women have the impression that there is a lack of understanding of their needs. Thus, the expected and positively perceived support is essentially material. The maternal grandmother was frequently mentioned and her support was generally perceived as positive. Nevertheless, midwives play a major role during this period, particularly for breast-feeding difficulties, and they are considered as a reference for decision-making. |
|  | The qualitative design of this study enabled the identification of sources of perceived stress experienced by primiparous women during the early and late postpartum periods, and insight into the coping strategies used. It also allowed the exploration of mothers’ perceptions of the social support received and its adequacy to meet their expectations during this sensitive period. In our study, the factors associated with postpartum stress are not major life events, but daily hassles. In the early postpartum period, women reported the interaction with the caregiver, followed by breast feeding and the hospital, as important stress related factors. During this period, social support is mainly provided by health  professionals. In our sample, women expressed their dissatisfaction with the support provided, both on the emotional and self-esteem levels. In contrast, an open and non-directive informative support was highly appreciated and expected regarding care of the infant. Sarason and Sarason (1990) noted that the effectiveness of the social support depends on the adequacy perceived between a type of support and its source. Caregivers should be more aware of women’s expectations in order to provide effective and adequate care during hospital stay, which has become markedly shorter in the last decade. The socio-cultural context may affect the type and importance of the stressful events. In a study conducted in Taiwan, the problems perceived as most stressful at three weeks postpartum were tiredness, lack of time and infant feeding (Hung, 2001, 2005). In our study at six weeks postpartum, the main stressful events were breast feeding, practical organisational aspects and the baby crying. Women highlighted major organisational difficulties, particularly when the family was absent or if the social network was perceived as unavailable or inadequate. The mother’s socioeconomic status may also influence the importance and impact of events. In our study, mothers were mainly from the higher levels and the categories presented in Table 2, as well as their frequency, need to be cross validated with a wider and more varied sample. Breast feeding as the major stressful event is an interesting and surprising result. In our study, 98.3% women were breast feeding when they left the maternity unit. Similar rates were shown in the same hospital by Boulvain et al. (2004). Our research showed that women perceived breast feeding as highly negative and threatening during the whole study period, with pain as the most important negative feature. Great value was placed on successful breast feeding, sometimes considered as one of the indicators of a ‘good’ mother. Women emphasised the discrepancy between the information given during the prenatal classes, which idealised breast feeding, and the actual reality. Today, breast feeding is actively promoted by society and professionals. Two metanalyses were conducted in this field. The first showed that structured information given during the antenatal period improves the rate of breast-feeding implementation (Fairbank et al., 2000), and the second that an educational intervention carried out in the prenatal period significantly improved the breastfeeding starting rate (Dyson et al., 2005). Our research highlighted the disillusionment of mothers when they observed the gap between their expectations and the difficult reality of breast feeding. |
|  | Although our interviews were centred on data collection, they did appear to have a beneficial effect on the well-being of participants, and this suggests a clear role for educational sessions during this period to improve mothers’ quality of life. In addition, our study raises concern regarding the information provided on breast feeding at prenatal classes, which clearly mitigates for a more realistic presentation of norms and expectations. Finally, our study was conducted in a sample of low risk, primiparous women in a West European urban population, and our results must be considered with caution and cannot be generalised to other settings. |
| Recommendations and  Conclusions | These preliminary results require further research to investigate and develop more effective methods to meet mothers’ expectations.  This study provides useful information for health professionals to reconsider postpartum care and follow-up. The findings highlight the discrepancies between the stressful events perceived by women, and the lack of adequacy between received and expected support. In the early postpartum period, the interaction with the caregivers and the hospital environment are the major stressful events. Although professionals are skilled in informative support, the expected support is more on an emotional and self-esteem level and the institutional environment should be aware of these aspects in the organisation of care. Breast feeding is a particularly stressful event. The difference between expectancies, ideal representations and the reality of breast feeding emerges as a real problem. The effectiveness of antenatal education on breastfeeding and postpartum events through informative support is not successful and it must be revisited and sustained postpartum. We highlight also the importance of the role of midwives in reassuring new mothers of their skills and the need to avoid provoking any feelings of inadequacy or guilt during this sensitive period. Coping strategies developed by women during the postpartum period are not specific. The lack of professional, long-term, postpartum follow-up is criticised by most women and further research is needed to promote and develop adjusted and effective postpartum support. Our study suggests that it is important to study the experiences of the mother, child and close family circle during the postpartum period. Stressful events during the early postpartum period are numerous and lived intensively, often negatively. Available social support for women is not always perceived as sufficient or adequate to calmly manage the different events occurring during this time. Knowledge and theories dispensed by health-care professionals are not always used or even considered feasible in practical terms by women. |

| 5. Title | Do contemporary social and health discourses arouse peripartum anxiety? A qualitative investigation of women's accounts |
| --- | --- |
| Publication year | 2015 |
| Author | Rowe et al. |
| **Participant Characteristics** |  |
| Participant group: Pregnant women/mothers (of children up to one year) | Mothers of infants up to 12 months of age |
| Number of children | First time mothers (12), two children (6), three children (2) |
| Age | 29-42 |
| Ethnicity | not given |
| SES | The participants were on average more socioeconomically advantaged than the general population of women who have recently given birth in Victoria |
| Recruitment strategy | With the assistance of the MPHMBU Nurse Unit Manager, all English-speaking women whose scores on screening instruments indicated that they were experiencing elevated anxiety but otherwise well, were given oral and written explanations of the study and invited to participate during the admission process |
| Sample size | Twenty women participated in one of four one-hour discussion groups |
| Eligibility | All English-speaking women whose scores on screening instruments indicated that they were experiencing elevated anxiety |
| **Methods** |  |
| Study aim | This study aimed to investigate the sources of worry and anxiety that women identify in the perinatal social and health milieu, the language and contexts they use to describe them, and the meaning that they ascribe to their experiences. |
| Theoretical background | not given |
| Design | A cross sectional design was used. Quantitative data were collected about participant characteristics and severity of symptoms of depression, anxiety and stress. The study was designed to investigate a phenomenon that had not been documented in this setting and there were no pre-specified hypotheses. Qualitative methods of collection and thematic analysis of data were therefore appropriate. |
| Study setting | The study was conducted at Masada Private Hospital Mother Baby Unit (MPHMBU) |
| Data collection approach | Discussion groups |
| Interview topics/focus | 1. Sources of worry about pregnancy and motherhood experiences 2. Thoughts, feelings, and behaviours that lead to excessive anxiety |
| interview/focus group duration | Four one-hour discussion groups |
| Use of additional resources/prompts | Prompts (such as “could you say more about that?” or “are there other examples?”) were used to promote elaboration |
| Analysis | Thematic analysis of the whole dataset was conducted using the group as the unit of analysis |
| **Findings** |  |
| Theme | **Themes— social and health discourses: Image management** Pregnancy is inherently anxiety-arousing. The data revealed feelings of uncertainty and vulnerability that were near universal. “When you are for the first time pregnant you are so unsure about what is happening, you are so unsure, is the baby OK? Is anything going wrong? (…) and there is so much anxiety (G4). Conception, pregnancy and infant care in high income countries are characterised by unprecedented medical and social surveillance, and prescriptions and proscriptions of many kinds about lifestyle and behaviour (Lupton, 2012). Acute awareness of constant medical and social scrutiny is connected to notions of the ‘good mother’ ideology of selflessness, prioritisation of foetal and infant needs and personal responsibility to protect the foetus and infant from harm (Wright, 2001). However, attribution of total responsibility for the health of the baby promotes excessive self-scrutiny and anxious preoccupation with personal behaviours. Both were potent sources of anxiety. But I was like whatever I am doing, whatever I am eating is going to affect my baby, I am an aerobics instructor and I was doing four classes a week from before I was pregnant until 39 weeks and I used to think “he is going to have a heart condition” and, you know, if he does it is my fault because I have been doing classes during the whole pregnancy … but in the end it's anything, everything you do, not having alcohol, the food, everything, you know, everything, and you think “this is crazy” (G3). Medical and social surveillance starts before conception, is solely focused on the woman and apparently ignores circumstances in her social context. Preconception—he [partner] thinks it is about you … the focus is on you, you have to look after yourself, and what about him? (G3) Surveillance continues through pregnancy. The pressure to delay disclosure of the pregnancy for fear that it will not progress was particularly strong when the pregnancy was the result of assisted conception. I didn't have IVF but I had to go through ovulation stimulation, it is extreme but not as IVF, so then I finally got pregnant and then to wait until 12 weeks, so those 12 weeks, and plus the risk for miscarriage as well until 12 weeks (G3). The anxiety associated with excessive personal responsibility for an outcome continued into the postpartum when it went beyond health to the behaviour of the infant. Even when I go to mothers group, I would prefer to hear the bad experiences and how they deal with them, because … I feel very bad because it seems that my baby is the only problematic one (G2). Heightened sensitivity to the opinions of others is a hallmark of anxiety. The anxiety aroused by excessive self-scrutiny was exacerbated when accompanied by perceived or actual inspection by others. I felt bad for her [baby] because it was so hot, and then you get more stressed and don't know what to do, and you have all these eyes on you and get more stressed (G1). The corollary of undue personal responsibility was guilt. I feel constant guilt for everything because even if she starts crying and I don't know why I feel like I must be doing something wrong and I have made her unhappy (G3). However, an understanding of the source of social pressure can help to re-frame these thoughts and alleviate distress, as this participant articulated clearly. When you go to mothers’ group you don't need to feel you are on top of the class … you know, it ends up being a competition of how fabulous [it is] and no one is having any problems (G2). Intolerance of imperfection leads to the drive to promote an image of the ‘good mother’ and underpins lack of confidence and symptoms of anxiety |
|  | **Breast is best** The “Breast is Best” message is regarded as central to promoting and maintaining high population prevalence of breastfed infants (Snyder, 2007). The single message was inescapable. In the media, every publication, every pamphlet, every website, just raises a norm about it [breastfeeding] (G1). Women's accounts demonstrated that they adopted this discourse uncritically. I understand that breast is best, but the pressure, I knew that there was going to be pressure, but I didn't know how much pressure … I don't remember the first weeks of [baby's name]'s life as a result, so yeah (G1). For many women this manifested as a preoccupation with feeding and overwhelming anxiety about failure. My son is five months and it [adequacy of milk supply] has been my major obsession of my life since he was born (G1). Embracing the “Breast is Best” discourse without question led to perceptions of guilt, worry and lack of confidence. There is a lot of guilt and shame in that, I have found that too, I would feel very guilty if I stopped, that is why I am still going (G1). The importance of the breastfeeding message to maintaining public awareness of the value of breastfeeding is indisputable. However, there is little countervailing discourse that gives a realistic appraisal of the risk of not breastfeeding and reassurance for women who do not breastfeed. I don't know anything of this [bottle-feeding], so we had to find out and rush to learn about formula feeding … and perhaps if they had given us a pamphlet it would just have been so helpful (G1). The health professionals' explicit and implicit endorsement of breastfeeding led to inequalities in health care for these parents. She [nurse] said “are you still breastfeeding?”, “yes” and she was “oh great, fantastic” and my poor friend was, and I just turned around to her and said “don't worry there is nothing wrong with it [bottle feeding]” and even when it wasn't directed to her, but the positive like “yes you are doing right you are breastfeeding” and I thought how horrible for her, this is actually indirectly making her feel she has failed (G1). The provision of non-judgemental reassurance that was missing from this account is an essential means of countering fears of social disapproval and of containing anxiety in new mothers |
|  | In the case of sudden infant death, the potential adverse consequences of the whole-of-population approach are exaggerated perceptions of risk of infant death and arousal of anxiety. Data revealed that women were aware of the SIDS public health discourse and many acknowledged that they knew that individual risk was quite low. Yeah, I know sort of rationally that it is quite low but probably the message is that it is higher than it is, I guess (G1). Nevertheless, there was widespread anxiety because of the ubiquitous messages about the threat of infant death. No, the fact is that it is very unlikely to happen… but yeah, I think that sometimes you can be a little bit overwhelmed by all the stuff that has to do with SIDS (G2). Other accounts revealed how perceptions of high risk reinforced catastrophic thoughts and unrealistic cognitions about personal responsibility for an outcome. All of these risks should be fully explained to people, but I was completely anxious … you don't realize how much those active messages might make you quite overly anxious because you think … if she is sleeping well I just go and check, my husband he is completely… he is worse than me he is completely paranoid about SIDS and we have got one of those monitors … (G2). Provision of nuanced information about magnitude of risk, importance of other risk factors, or whether the risk is the same for all families, might be counter-productive to the goal of a social marketing campaign. However, omission of balanced information led unintentionally to feelings of fear and inadequacy. Just offer me alternatives that are also valid so at least I don't feel that I am doing a really bad job because I cannot maintain my baby on the back all night or I cannot breastfeed my baby, just give me alternatives (G2). An orientation to threatening information is characteristic of anxious arousal. Participants repeatedly called for sensible information that describes the problem and its solution in realistic terms. A more balanced message could ameliorate experiences of social disapproval, enable freedom of choice, ensure appropriate clinical care, and ease anxiety (Shah, 2013). This study was conducted when new advice about the risks to infant safety of bed-sharing was disseminated publicly (Raising Children Network, 2012). There are ambiguous theories … I had my baby in bed from pretty much her birth yeah, some say now co-sleeping is [unsafe] … but when I had her months ago, I read that cosleeping was safer (G1). Inconsistent information about such important matters was deeply unsettling and reinforced anxiety that was underpinned by intolerance of uncertainty about the right thing to do. Messages that were difficult to implement led to excessive fear and guilt. You know I got into the habit of co-sleeping with her…she would fall asleep after a feed … you freak out and in, you know, 45 minutes later … my husband is saying “you got to put her back in the bassinette, this is just not safe, you know, I'll show you the latest research saying that co-sleeping is not good” (G2). Some public messages are sensational and alarming. An American campaign used a graphic depiction of threat on a poster showing two babies, each sleeping in an adult bed, one beside a cleaver and the other beside a kitchen knife. The caption reads “Your Baby Sleeping With you can be Just as Dangerous” (Llorens, 2011). Thoughts of infant death are inherently distressing; sensational health messages are therefore unnecessary as well as potentially damaging  **Just speak up** Australia's national mental health campaigns (beyondblue, 2015) have led to increased public awareness of depression, including in the perinatal period (Highet et al., 2011). The ‘Just Speak Up’ campaign concerns postnatal depression specifically. Encouraging women to express their emotional problems and seek professional help is regarded as a worthwhile goal of the campaign. However, there is also a danger of pathologizing normal adjustment reactions and creating confusion and fears about negative evaluation and personal vulnerability. Women's accounts revealed their awareness of the hazard of postnatal depression but a view that the label can be over-used. This participant described feelings of vulnerability, stigma and defensiveness aroused by the spectre of postnatal depression. There is this whole focus on postnatal depression …yes, but if you have that don't feel bad it is not a sign of weakness, it doesn't mean that you are going around the twist … it's OK to feel these anxious things … you know, it's just tiredness, I don't have postnatal depression (G2). Australian national perinatal mental health recommendations include routine screening for depression symptoms in primary care and referral for treatment (beyond blue, 2011). The potential for mass screening programmes to provoke anxiety is well described, a cost that is regarded as outweighed by their benefits for population health |
|  | In the perinatal period, EB practice can be confronting, because of the large volumes of relevant information, the frequent need to make decisions, and because the woman is required to act autonomously in the context of her growing attachment to and responsibility for the wellbeing of her child. This participant felt overwhelmed by the need to incorporate a lot of new information. There is so much stuff, I had to say to the nurse “can you repeat all that?” as a first-time mum (G4). Despite this, discussions revealed a perception that individuals are expected to take personal responsibility for appraising information. Well, everything is online now, so they probably expect people to educate themselves (G3). This is despite the fact that decision making operates in a world of imperfect information and every website is different (G3). When interpreting evidence, clinicians and individuals must make decisions that are in the best interests of an individual. However, this is usually based on epidemiological evidence that is relevant to populations but not individuals (Haynes, 2002). One woman described amelioration of her anxiety when she was relieved of the responsibility of making decisions herself. And I am not a professional, I don't really know how to handle this and he (obstetrician) was like “this is what is going to happen”, and that sometimes is more reassuring than you having to come up with your own opinion (G3). Coinciding with this shift of emphasis to EB practice is the explosion in the amount and ready availability of information as these participants experienced. I was overloaded by too many things (G1); I got on the internet way too much (G2). Free online access to high volumes of information of variable and uncertain quality created the immediate opportunity for anxious and repetitive searching for reassurance and to avert doubt, which characterises and can exacerbate anxious arousal.  **Risk society**- Health psychology evidence suggests that communicating the risk conveyed by a screening test result without raising anxiety may be facilitated by using numbers rather than words (Marteau et al., 2000). However, this might not apply to someone experiencing anxiety-induced cognitive confusion or using an avoidant means of managing anxiety that is driven by the need to reduce uncertainty, as this participant described. And I think, it would have been better not knowing the number, it would have been better for them to say to me “you are in a safe category” rather than “your child got …” (G3). Further, perceptions of risk are subjective. They are influenced by the available information, as well as how probabilistic and threatening information is processed (Marteau & Kinmonth, 2002). Information processing is affected by perceptions about the likelihood of an event, but cognitive and emotional processes determine how a person frames the potential cost or threat of the event. This participant framed the threat of losing her much desired baby as greater than that of giving birth to a baby with Down syndrome. And, yeah, the whole thought of doing all of that [assisted conception] and then have a diagnosis of Down syndrome and being faced with that decision, I don't think I would have terminated it (G3). It is known that even when told that the risk is “low” or the result is “normal”, some people have difficulty discarding the residual risk that is inherent in a screening test result and continue to regard themselves (or their child) as at risk (Marteau, 1989). Because I did have that you know, result and after a while it was OK, but I must say that the first few weeks after the result I was a little bit, just not so excited as how I was with [baby's name], where everything was fine, you know, it wasn't the same but then … it was just fine (G3). This participant described lingering anxiety about a “normal” test result |

| 6.Title | Pregnancy-Related Anxiety in Women Who Conceive Via In Vitro Fertilization: A Mixed Methods Approach |
| --- | --- |
| Publication year | 2016 |
| Author | Stevenson et al. |
| **Participant Characteristics** |  |
| Participant group: Pregnant women/mothers (of children up to one year) | Pregnant women between gestational weeks of 12 and 18 who conceived using IVF treatment |
| Number of children | not given |
| Age | 25- 40 years |
| Ethnicity | White (74.1%) Asian (16.2%) and Black or African American (2.1%) |
| SES | The sample was highly educated and affluent, with 85.5% having at least an associate’s degree, and 69.7% reported household incomes of $100,000 |
| Recruitment strategy | Subjects were recruited from a private infertility practice in Northern New Jersey, which performed approximately 2,700 cycles of IVF during the time of data collection |
| Sample size | Thirty-one participants provided narrative data about their pregnancy- specific anxiety |
| Eligibility | Women had to be between the ages of 25 and 40 years, be pregnant with one or two foetuses, be between gestational weeks of 12 and 18, and have the ability to read and write English. They were excluded if they had selective reduction in the current pregnancy or were deemed medically or obstetrically high risk. |
|  |  |
| **Methods** |  |
| Study aim | The aim of this study was to measure pregnancy-specific anxiety quantitatively and evaluate this anxiety qualitatively in women pregnant via IVF using a mixed methods approach by describing the level of pregnancy-related anxiety in women pregnant via IVF during early second trimester, and to identify themes in anxiety specific to pregnancy. |
| Theoretical background | not given |
| Design | A mixed methods study |
| Study setting | An infertility practice in Northern Jersey |
| Definition/conceptualisation of stress and/or anxiety | not given |
| Data collection approach | Semi structured interviews (open ended questions) |
| Interview topics/focus | Women’s experience of anxiety related to the pregnancy |
| interview/focus group duration | not given |
| Use of additional resources/prompts |  |
| Analysis | Content analysis was used to organize and integrate data from the comments into themes or patterns |
| **Findings** |  |
| Theme | **Theme 1**. The most common anxiety reported by patients was regarding the health of their unborn baby(ies). Twelve of the 31 subjects cited having this as their additional anxiety, using such words as fear, worry, anxiety, and stress. “Nothing really specific . . . just after everything we went through, there is always an underlying fear that something will happen to the baby . . . I don’t think I’ll relax till he/she is here.” Another stated, “Just scared that the baby is not okay.” Some women continued to think about the health of their baby even when not awake. “Dreams that are about me being nervous or things not going correctly.” Several subjects cited their older age as affecting the anxiety about the health of their baby, specifically mentioning upcoming genetic testing. “I would say the biggest stress I’m having is my upcoming amniocentesis. It’s happening at week 16 and I’m worried my results won’t be normal. My age is a big factor in the odds of having a baby with a genetic disorder. My age is higher because I couldn’t get pregnant. It seems unfair that you go through the emotional and physical stress of fertility treatments, only to get the outcome you desire, only to continue to be stressed about the health of the baby. Seems like you just trade on stress for another, I’m anxiously waiting to feel calm and excited about my baby, rather than concerned about his/ her health because I waited too long to conceive.” |
|  | **Theme 2**. A second theme that emerged was related to the woman’s perception of her own health and safety. Six subjects reported having this anxiety. “I am very worried about my recovery after childbirth and the postpartum period.” Some of these anxieties were specific to self-care and making sure their body was as healthy as possible for their pregnancy. One subject reported having an anxiety about “Appropriate weight gain: too much, too little, when it happens, etc., eating the right foods, getting the right nutrition." The changes of pregnancy can often be an adjustment for some women. One woman stated: “I worry about every single ache and pain. I wish I could enjoy my pregnancy." |
|  | **Theme 3**. Another theme that emerged from the data was about a woman’s perception of her own abilities in the role of mother. Three subjects cited this as an additional anxiety they were experiencing. Sometimes it is related to the new experience, having not had a child before and not knowing what to expect. One subject reported having anxiety: “That I will not be able to properly care for my first child.” In another instance, multiple gestation was a significant contributor to anxieties related to abilities to parent. “Having twins is making me anxious about my ability to care for them as well as my daughter who will be 3 years old at their birth.” |
| Discussion | This study advanced previous evidence on anxiety among women who conceived their pregnancies with IVF, further identifying an increase in this population. It also expanded the reasons for this anxiety via the qualitative results, allowing a richer understanding of this experience. Despite the fact that this study sampled during the second trimester which is normally associated with the lowest levels of anxiety during the pregnancy, this population still had elevated levels and articulated specific anxieties they were concerned about. In addition, participants were also given the opportunity to complete study surveys from their home, which is in contrast to most other studies that data collect within the setting of office visits, which can impact anxieties. This suggests that this sample was at risk for greater anxiety at other points during their pregnancies, particularly at the beginning of the pregnancy when the pregnancy’s future is less certain or toward the end as childbirth approaches. This adds to existing literature and illustrates the need to examine this concept longitudinally in future research. The other objective of this study was to identify themes in anxiety specific to pregnancy. Qualitative themes that emerged helped expand and enhance the data from the PRAM by providing underlying meaning, emphasis, and nuance beyond what is obtainable from quantitative measures. These themes included anxiety about the health of their unborn baby, their perception of their own health and safety, and their perception of their abilities in the role of mother, which will be discussed specifically in the following paragraphs. The first theme to emerge was related to their anxiety about the health of their unborn baby(ies). Previous research reported that many women felt that the IVF pregnancy might be their only pregnancy, were “fixated” on it, and thinking about it incessantly. For these women, anxiety about their baby’s health often manifested itself in disrupted sleep, increased nightmares, and compulsive behaviours such as constant checking for vaginal bleeding. Although positive foetal cardiac activity at 6 weeks’ gestation was found to be an excellent predictor of first-trimester outcomes in IVF pregnancy (Seungdamrong et al., 2008), only during certain milestones such as movement seen on ultrasound did this anxiety temporarily ease (Hjelmstedt, Widström, Wramsby, & Collins, 2003). Women in this study expressed angst about their anxiety, wishing for a more sense of ease so that they could enjoy the pregnancy which had taken so long to achieve. The second theme to emerge from this data was anxiety about the woman’s perception of her own health and safety. With such intense focus on their reproductive function, this population understandably expressed this concern. Moreover, women who have increases in pregnancy-related anxiety and specifically fear of childbirth may not be able to prepare properly for their birth (Rouhe et al., 2013), and if they have not made adequate preparations or be fully informed of the process and what to expect, they are more likely to be emotionally vulnerable during childbirth and be unprepared for certain obstetrical procedures and complications. Perception of women’s inadequacy in labor or control over their health was found in previous research (Bryanton, Gagnon, Johnston, & Hatem, 2008). Collectively, these anxieties can have a significant impact on the birth process. For example, women with high levels of fear of childbirth significantly increase their use of epidural anaesthesia during labor (Hall, Stroll, Hutton, & Brown, 2012) which can increase risk of other potential negative outcomes. In addition, other research has found a correlation between prenatal anxiety and increased rates of caesarean surgery (Zhou & Li, 2011). The third theme that emerged from the data was the anxiety about a woman’s perception of her own abilities in the role of a mother. The few days following birth are a critical process of maternal role attainment (Mercer & Walker, 2006; Rubin, 1967), and stress has been found to influence this process (Mercer, 2004). The challenging journey required for a woman to achieve IVF pregnancy may contribute to questions about her capabilities as a mother. This may be a common theme for all expectant mothers—but in this population, the stakes are likely to be higher to be that “perfect parent.” Perinatal nurses, providers, and doulas caring for women in both the days and weeks following birth should be in tune with this adaptation process and make appropriate recommendations |
|  |  |
| Conclusions | In conclusion, throughout the journey from IVF through birth, a woman may understandably have increased anxiety. Although additional research is necessary to further understand stress and anxiety during pregnancies conceived by IVF, this study has provided insight about the experience of anxiety during the pregnancy and has identified specific anxieties mothers experience about the health of their babies, themselves, and their perceived ability to parent |
| Recommendations | Future research should examine this stress longitudinally and explore mediating and moderating variables, as well as ascertain potential interventions targeted at reducing anxiety in this population. |

| 7. Title | A qualitative study of pregnancy-related anxiety among women in Tanzania |
| --- | --- |
| Publication year | 2017 |
| Author | Rosario et al. |
| **Participant Characteristics** |  |
| Participant group: Pregnant women/mothers (of children up to one year) | Pregnant and postpartum women obtained high scores on a PRA scale during pregnancy in a larger quantitative study |
| Number of children | not given |
| Age | 18-34 |
| Ethnicity | not given |
| SES | Mixed |
| Recruitment strategy | The study sample was obtained from a quantitative study underway to examine the individual and collective contribution of PRA and depression in the prediction of preterm birth and postpartum depression |
| Sample size | 10 women |
| Eligibility | Women (a) 18–34 weeks’ gestation, (b) able to speak English or Swahili and (c) with high scores on the 10-item PRA scale,14 which assesses foetal health, loss of foetus, childbirth, mother’s well-being, parenting and control/ confidence. |
| **Methods** |  |
| Study aim | To explore and understand the experiences and priorities of pregnant women living with fears and worries related to foetal/infant and maternal health, the birthing process and ability to parent the infant (i.e., pregnancy-related anxiety (PRA)) in Mwanza, Tanzania |
| Theoretical background | Not given |
| Design | A qualitative study design using semi structured interviews |
| Study setting | Clinics in Mwanza |
| Definition/conceptualisation of stress and/or anxiety | Pregnancy related anxiety is a syndrome that is distinct from depression, stress or generalised anxiety |
| Data collection approach | Semi-structured interviews |
| Interview topics/focus |  |
| interview/focus group duration | 40-75 minutes |
| Use of additional resources/prompts |  |
| Analysis | Descriptive phenomenological approach |
| Rigour (Saturation, reflexivity) | Repetition of data that had been previously collected was achieved after 10 interviews, indicating data saturation |
| Theme | **Knowledge and understanding:** Lack of knowledge, or understanding of what was normal, was an underlying issue in many of the worries participants had about pregnancy and taking care of a new baby. Berta disclosed: I get frantic at times because I know that my knowledge and understanding of what a baby might be suffering from in low. Currently she is active in my belly. But when she is born, she might cry at night and there is no one to help me learn what might be wrong with her. Mostly I will guess. But for the days and nights now, I worry what could happen to her because of me. Margaret found herself asking people around her about symptoms she was experiencing. “For instance, last week I had very severe abdominal pain for about 3hours; I could not sleep. People tell me that it is a normal situation". Neema experienced heightened anxiety as she could not understand her somatic symptoms explaining: I felt bad and I was ill. About my health, after seeing myself very thin since I was so often ill, though it (was) for all my pregnancies but in this one it was severe with a lot of stress…The felling of body weakness and fatigability went throughout the pregnancy. Both Neema and Margaret experienced symptoms that they feared might be attributed to HIV and were terrified that this might be the cause of their symptoms. Margaret ‘fail(ed) to eat’ and was ‘vomiting a lot’, while Neema ‘felt ill’, became ‘very thin’ and was overcome with ‘weakness’, all of which contributed to the fear of having HIV. Neema explained, “I was tested when I was about 8 months pregnant for HIV infection and I was fine, I was not infected. But all those months, I thought I was". |
|  | **Partner relationships** Grace described the support her partner provided after she and their first child was diagnosed with HIV as, "(he) didn’t disturb or humiliate me as for why he has no AIDS and we do, he accepted us". Grace kept on thinking of the current pregnancy “if all my children will be infected how things would be. Even when I rest for a few days, about 3 or more days then the concerns keep on coming back". Neema candidly shared the worries caused as result of her husband: I don’t know what the issue was but they say some might hate you when you are pregnant, I thought that was the reason. When you are in such situation and he comes asking for sex, it is an issue. It was very difficult for me…maybe because he found me often sick, maybe he was hurting inside…happiness diminishes, love fades away. I thought he might have been seeing another woman. I don’t know, but at the late stages of my pregnancy we became close, he changed and that was a relief. There were participants who had longed for emotional support from their partners but were unable to obtain it. Grace’s husband travelled frequently; she explained “as his work is that he has to travel…he is often not at home…he does not stay at home most of the time. So it is not easy to tell him what happens to me every day". Berta explained that at one point in her pregnancy, my partner took me to my parents’ place and left me there. No one would talk to me without my husband there. Even visitors would only talk to my father. It was not like they were coming for me, or to know how I feel or how I was doing. They had no time to ask all that. Conversations with research associates revealed that women in Tanzania (or Tanzanians in general) often do not discuss personal concerns with friends, or even family. Once Berta returned from her parents’ place, she explained ‘During that time (pregnancy) my husband was not around, he had travelled. We had poor communication when he was away. Whenever I called, he was not reachable. At the time he was away, I was already confused because (of) my situation and I needed him… but at the time I couldn’t get through to him, I was very worried during that time’. For participants in this study, the notion of support was contextually and culturally defined as financial support, as gleaned from peer debriefing. Aisha had moved to Mwanza from her rural hometown, where weeks passed since her partner left. Aisha had little contact with the father of her unborn child and received little help when he did respond. "What gave me worries was…there were times I…asked for support and he wouldn’t help me". |
|  | **Interactions with the healthcare system** All participants in this study received antenatal care at least once during pregnancy. Margaret shared "I am used to calling the nurse whenever I get worse, I meet her and she helps me". Margaret would ‘do whatever the doctors instructed (her) to do’ and found “I feel peace whenever I go to the hospital, since (she) gets advice from them to be well and keep (her) baby well". Joyce also recalled positive interactions with healthcare providers; however, “what I see is, they (health care providers) are more supportive in this pregnancy, especially the nurses. Whenever I face a problem they advise and help me". However, some women experienced less favourable interactions. Berta recalled a stressful week in which numerous providers contradicted her diagnosis of ‘low blood’ (a common term used to mean anaemia—often women will know this phrase in English without the need for translation) and informed her "that I might fall down or deliver a dead baby or that is how all of my reproduction will be". The worry in her voice and the expression on her face depicted even more than her words. Her voice grew shaky, her eyes began to water and she fidgeted with her hands in her lap. Berta felt as though the interaction with healthcare providers had exacerbated her worries explaining: So she didn’t give me clear information or encourage me. I felt like she was adding on my problems since when I was trying more to get information from her, she said 'There is this lady who came here with the same situation as yours’; I directed her, so let’s find her, she can give reliable information. So herself as a nurse who was just advising me on my issue couldn’t know where I could get help. So I feel she added on me a load in my thoughts. Neema described a particularly difficult day in which she had arrived very early at clinic for assessing high blood pressure. Reflecting on the process of taking number to receive service she shared: I came early and I was ill on that day. I was the fourth lady to arrive. At a time we were provided with numbers I never knew, I had gone to the toilet and there were about 99 women in queue. I missed the number. The nurse refused to give me a number saying ‘you were not around, there are people who were here early, you came late and you want number'. I shed tears, I was upset and sad, I felt bad and I was ill, I went into the room where they take physical measures. Sarah had waited a long time to become pregnant thus when the time finally came, “I wished to see my stomach grows fast. I used to check up on myself every now and then I felt like my stomach was not growing, so I felt worried…if the child was alive inside my womb". Sarah described frustration in being advised to stay on bed rest after experiencing bleeding in the first trimester. She stated, I don’t want to take the bed rest, though they advised me to, I figured out that I couldn’t because I am working…I don’t have much time to stay at home (considering that) I would have to take maternity leave after delivery. I was so worried, I was frustrated. That was very hard for me. While routine prenatal care was provided free of charge in Mwanza,29 strained relationships with healthcare providers, the inability to access additional services or lost time at work, was an underlying issue in many of the narratives |
|  | **Spirituality** The women’s spirituality permeated many conversations, often woven among the fears and concerns they expressed. Imara had three girls and felt worried as her sister-in-law’s wanted their brother to marry another women who may be able to give birth to boys. She explained how her belief in God helped her to ‘take heart’ (i.e., took courage) by thinking: God is the one who provides, it is not like I am going to the market, that I choose I want this and that, no, I encouraged myself that God is the provider you can’t say that I have only girls…I believe God knows more and I don’t. Wema’s sister-in-law died from pre-eclampsia. When asked how she managed to cope with her worries, Wema simply replied, “I only prayed to God…I just pass through; it reaches a point I had to take heart, thought it was God’s plans". Similarly, Berta believed that ‘God is the one who knows it all…' including her own future and if she ‘will deliver safely or not’. Despite her struggles, Berta ‘was encouraged by being thankful to God in everything'. Sarah had experienced particular anxiety that her stomach was not growing large enough to indicate that a healthy child lay inside. For Sarah aside from her husband “Honestly, my other help was from prayers only. When I pray I get peace of mind. I stopped worrying. I believed my stomach would get larger. So as much as we talked and prayer, I felt peace". |
|  | **Fears of HIV/AIDS:** consumed the thoughts of the pregnant women who could not explain their somatic symptoms. Margaret explained that, “I was vomiting a lot, not eating well, it made me have poor health and I became very thin. So I was thinking, I had not tested (for HIV) and I was very worried". She described feeling scared “after seeing myself very thin, though it (was) for all my pregnancies but in this one it was severe with a lot of stress". Neema, who also saw herself feeling thin and unwell, described, it was during my early pregnancy but the feeling of body weakness and fatigability went throughout the pregnancy, I was tested when I was about 8 months pregnant for HIV infection, I was fine, I was not infected. My worry was because I was often sick and had lost weight so much, so event that added in my thoughts. The three HIV-positive women had relatively recent diagnoses (within the past 3 years) were preoccupied with worries about their unborn child/infant, and concerns for their child’s future if they were to pass away. Grace talked about her reactions to her HIV-positive diagnoses: I was frightened and full of worries. I passed about three days without food since I had a lot of stress and thoughts, I was sad, I was worried and I cried so much. I never expected my baby be infected or even myself; I never knew until I became pregnant again and I found out that I was HIV infected together with my oldest child. Grace also discussed her concerns about the antiretrovirals that she began taking during her pregnancy and their impact on her unborn child. She explained, those drugs were bringing me different conditions (side effects). I felt like dizziness and was thinking was these drugs to protect the child who was in my womb? If they make me feel this way, and in other days, I even lack sleep, with these conditions will my child be alive? Will the drug help me? I was worried. Joyce’s initial "concern was perhaps my child would also get infected, I asked the doctors, they reassured me and I stopped worrying". However, the initial reassurance faded as she considered her own diagnosis and then explained, “Both of my parents died and relative from my husband’s side know nothing…I am worrying of delivering a baby who is HIV infected and was wondering what if I die early, who would I leave my baby with?" |
| Discussion | The PRA scale14 has been identified as an appropriate tool for examining the experience of PRA for women in high-income countries. Nonetheless, there have been claims that this tool, along with others designed to examine this phenomenon, displays relatively narrow domains that may not reflect the essence of PRA experienced by women. Our study confirmed this assertion for women in LMIC, specifically in Mwanza. Lack of knowledge, partner relationships, interactions with the healthcare system, spirituality and fear of HIV/AIDS are suggested as additional domains based on their strong presence in the narratives and themes derived from the participants’ stories. Table 1 provides suggestions for associated prompts related to each of these domains based on the participants’ stories. Together with the PRA scale, these prompts may assist in developing a comprehensive measurement scale that will more accurately depict the sociocultural context of life in LMIC, specifically Mwanza, and therefore more accurately assess and identify pregnant women who are experiencing PRA in this region. The literature on PRA2–9 outlines the numerous adverse effects for both mother and baby; many of these consequences of PRA were echoed in the participants’ narratives. Participants appreciated that ‘thinking too much’ or worrying was bad for them, as well as the baby. Partners/husband, friends, family, neighbours and care providers informed these sentiments and "advised that the more you keep on thinking, the more you risk your baby" or you "might get miscarried or bear a child with weakness…don’t stress, you might worry much and lose your life or the baby’s". The stories of family, neighbours and friends become increasingly valuable as a source of information due to perceived lack of knowledge of healthcare professionals or lack of trust in their advice. In LMIC such as Mwanza, the use of the word support was complicated by poverty, as often fathers/partners may continue to provide financial resources, exacerbating a woman's dependency for survival, while providing little to no practical or emotional support. There is increasing evidence that mental health issues in women may be related to social circumstances, including poverty, violence and economic dependence. The stories of the women in this study are similar to previous studies that indicated the significance of financial supporting women’s feelings of anxiety. Mwanza, and Tanzania in general, remains a highly patriarchal society in which women are often not given ownership of their decisions, including decisions regarding health and medical care. Research colleagues in Mwanza indicated that this might be due to any number of issues, including inability to meet sexual needs, decreased ability to provide for the home or the partner’s stress about providing for another child. For many participants, these sex-based realities of life influenced family roles, interactions and emotional support mechanisms and evoked feelings of frustration, anxiety, hopelessness and resignation. Lack of perceived control over a situation can affect an individual’s uncertainty and insecurity, often leading to increased anxiety. Nearly all of the women used spiritual-positive coping—religion, prayer or belief in a greater power or plan—to overcome distressing situations and their potential or actual negative consequences. Spiritual-positive coping offers a framework to help individuals make sense of the bigger life questions, placing the power of a situation on a greater entity. This can have many effects, including a reduction of guilt and feelings of responsibility for a difficult or unfortunate situation. Spirituality may lower anxiety levels, increase feelings of security, provide additional social support and reduce worry. Few studies have examined spirituality and mental health in Africa, despite it being recognised as a significant aspect of daily life in Tanzania. In LMIC, financial constraints and multiple socio-political problems (e.g., poverty and economic instability) that burden healthcare systems contribute to worry and uncertainty throughout pregnancy. For participants with access to care, the experience was at times disappointing due to negative events endured with healthcare providers. In Tanzania, antenatal services are widely available; however, quality of service and treatment remains an ongoing issue due to lack of trained personnel, inadequate supplies and equipment and poor implementation of antenatal guidelines. Three of the 10 participants were HIV-positive, yet HIV played a significant role in their stories and in the lives of many of the participants. Many non-HIV-positive participants expressed constant fear or anxiety about being diagnosed with HIV. Their worries did not indicate a positive diagnosis, but rather the reality of living in a highly affected area, with a heightened sense of the reality and risks of this illness |
| Conclusions | How social processes, poverty and culture informs mental health of women during pregnancy remains unexplored. The social, economic and cultural realities of women residing in Mwanza were exemplified in themes of lack of knowledge, partner relationship, interactions with the healthcare system, spirituality and fear of HIV/AIDS that emerged from the narratives of women experiencing PRA. Our study provides insight in this regard and adds new knowledge about the essence of PRA experienced by women in Mwanza, Tanzania. The PRA scale appropriately explicates participants’ fear, worries and concerns related to their own health and survival during pregnancy, their infants’ health during pregnancy and survival as well as caregiving ability in the postpartum. The PRA scale, however, narrowly reflects domains that inform our understanding of PRA of women in Mwanza, Tanzania and perhaps other LMIC. |
| Limitations | Due to time constraints, it was not feasible to return to the participants to present initial findings and collect feedback, as per Colaizzi’s method. |
| Recommendations | Not given |

| 8. Title | Themes of stressors for childbearing women on the island of Hawaii |
| --- | --- |
| Publication year | 1993 |
| Author | Affonso et al. |
| **Participant Characteristics** |  |
| Participant group: Pregnant women/mothers (of children up to one year) | Pregnant women and postpartum women |
| Number of children | not given |
| Age | 18-40 |
| Ethnicity | Japanese, Filipino and Hawaiian |
| SES | Filipino and Hawaiian (Low SES), Japanese (middle to upper SES) |
| Recruitment strategy | Recruitment was assisted by using a pilot survey of approximately 30 women which revealed women preferred to express themselves through focus group discussions |
| Sample size | Five focus groups with seven to ten people in each group |
| Eligibility | not given |
| **Methods** |  |
| Study aim | To describe themes of stressors reported by women living in rural communities known as East Hawaii on the island of Hawaii |
| Theoretical background | Not given |
| Design | A qualitative study design utilising focus groups discussions |
| Study setting | Rural communities on the island of Hawaii |
| Definition/conceptualisation of stress and/or anxiety | not given |
| Data collection approach | Focus groups |
| Interview topics/focus | Women’s feelings during pregnancy and events/experiences which are difficult for women to handle during pregnancy |
| Interview/focus group duration | 90-120 minutes |
| Analysis | The data is analysed using elements of an ethnographic summary approach and systematic coding via content analysis |
| **Findings** | Three themes were prominent for Hawaiian women. These women were preoccupied with concerns over their body image; overwhelmed by stress and conflict from the relationship with their spouse or mate who was perceived as dominating or exhibiting ''macho'' characteristics and felt conflict over their family’s tendency to be overprotective toward females beginning in infancy and extending to adulthood. Body image concerns were evident by frequent comments related to the following: getting fat and looking ugly, not being able to wear the clothes of one’s choice, thinking that one will still be fat after childbirth, being anxious about getting back into pre pregnancy -size clothes, and being upset at seeing pretty clothes that one can no longer wear. These concerns are similar to those expressed by samples of women from middle-class urban backgrounds. What makes this theme of stress an important finding for health professionals is that Hawaiian women typically convey an attitude of indifference about their body image. During standard prenatal care services, Hawaiian women are known to joke about their appearance and have been erroneously judged by health professionals as not concerned about their body image. This theme was repeated in every focus group session conducted with Hawaiian women. Possibly some Hawaiian women have acculturated to the modern American lifestyle through endorsement of an anorexia mentality that leads to cognitive distortions for childbearing women. The distorted cognitive paradigm is that fat equals ugly and pregnancy equals fat; therefore, a pregnant woman is fat and ugly. This finding about Hawaiian women concerns over their body image is important for tailoring prenatal care programmes to a more culturally sensitive direction while addressing women’s concerns and feelings about their body image to be more congruent with Hawaiian cultural beliefs and to become less attached to the American belief that equates fat with ugliness. Hawaiian women expressed concerns and discontent with certain aspects of the relationship with their men. Some Hawaiian women perceived their men as having a need to direct or control them through behavioural components that are not pleasing or satisfying. Women reported how they were made to return home and remove their makeup and jewellery or to change their hairstyle if their mates perceive them as looking too proactive. This finding indicates the need for providers of prenatal care services to work with expectant Hawaiian fathers. Hawaiian women wanted men to understand that their desire to look and feel good about their physical appearance throughout pregnancy represents their private struggles in coping with their changing body image. |
| Theme | Three themes reported by Filipino women were discomfort in seeking services outside their families and ethnic communities, preoccupation with stress arising from the need to gain social acceptance in the community and a cognitive style that influences wishful thinking, dreams and expectations throughout pregnancy that are not congruent with their lived experiences or the realities of their lifestyles. Pregnant Filipinas in Hawaii generally do not go outside their immediate or extended families or their ethnic group for help. They are a cohesive cultural group that employs elaborate mechanisms to provide resources for social, economic and environmental supports for each other and their ethnic group as a whole. This attitude permeates their behaviour towards health care. Health professionals frequently experience difficulties locating Filipinas in the community for the purposes of identifying their unique needs, recruiting them into health care services and monitoring their services when longitudinal services are necessary. Several factors contribute to these recruitment and retention problems. Filipinas tend to be shy and timid regarding the use of health care services and they rarely ask questions out of deference to health professionals. In particular, frequent use of medical jargon by health professionals is perceived as intimidating and offensive by Filipinas. Therefore, it is not surprising that Filipinas are often latecomers to prenatal care and are targeted as the ethnic group with no prenatal care. |
| Conclusions | Stressors arising from a rural lifestyle coupled with multiple ethnic and cultural belief systems that are often in conflict with the philosophies of available healthcare systems have yet to be addressed. Current prenatal care continues to be driven on the basis of a medical, bio-physical paradigm. Community health caregivers are challenged to work within women’s cultural belief and practice systems that provide resources for them to cope with life circumstances that become complex through pregnancy. The gaps that arise from a lack of understanding of rural and ethnic issues can be bridged by community health caregivers who are sensitive to conducting assessments that yield qualitative data on ethnic women unique circumstances during pregnancy. |
| Limitations | not given |

| 9. Title | I Am Pregnant and Want to Do Better but I Can’t: Focus Groups with Low-Income Overweight and Obese Pregnant Women |
| --- | --- |
| Publication year | 2015 |
| Author | Chang et al. |
| **Participant Characteristics** |  |
| Participant group: Pregnant women/mothers (of children up to one year) | Pregnant women |
| Number of children | not given |
| Age | Average age of 25.9 |
| Ethnicity | White (52) African Americans (44) |
| SES | Low income |
| Recruitment strategy | Participants were recruited via personal invitation at 4 collaborating Special Supplemental Nutrition Program for Women, Infants, and Children (WIC) sites in Michigan between May and August 2011. |
| Sample size | 96 pregnant women |
| Eligibility | To be eligible to participate in this study, individuals were required to have a pre-pregnancy BMI at least 25.0 kg/m2 (calculated using self-reported height and weight), be African American or non-Hispanic white, be at least 18 years old, speak and understand English, and be enrolled in WIC. |
|  |  |
| **Methods** |  |
| Study aim | The study aimed to identify factors that influenced stress, healthy lifestyle behaviours (healthful eating and physical activity) during pregnancy |
| Theoretical background | Social Cognitive and Self-Determination Theories |
| Design | A cross sectional design was used to conduct 7 focus group discussions |
| Study setting | not given |
| Definition/conceptualisation of stress and/or anxiety |  |
| Data collection approach | Focus group discussions |
| Interview topics/focus | Daily stressors, changes made after becoming pregnant, barriers and facilitators for eating healthier and being physically activity during pregnancy and knowledge of health in relation to gestational weight gain |
| interview/focus group duration | 2 hours |
| Analysis | not given |
| **Findings** |  |
| Theme | **Daily stress- Social Support**-Poor communication that influenced their relationship with significant others was frequently mentioned as a key stressor in pregnant women’s daily lives. Women were frustrated or upset with spouses/boyfriends for 3 key reasons—failure to understand or listen to women’s pregnancy concerns, refusal to be helpful when asked, and being overly concerned with the pregnant women’s safety. A woman said ‘‘It seems to me that men irritate you the most when you are pregnant cause like you do not understand what a woman feels like when she is pregnant… I wish God would let men carry a child and then ya’ll give birth’ ’Another one said ‘‘It seems like he’s the pregnant one and he’s acting so sensitive every time I do something. Now we are not talking’ ’Another stressor was that the pregnant women were told by many friends and family members what they could or could not do. Thus, the pregnant women felt a lack of personal control. A woman said ‘‘I think what stresses me out is when people try to tell you what you can and can’t do. You know ‘you don’t need to eat that.’ If you’re craving it, you want it; whether you supposed to have it or not, it’s just something you know when somebody tell you don’t need to eat that’’. Another one said ‘‘She’s (her sister) always worried about me so she’s constantly texting me, this is what you need to do, you need to eat this, you need to drink this’’. Many women said that taking care of young children at home was stressful. A woman said ‘‘I have a 10-month-old so it’s really challenging for me…trying to run after him and getting home all situated it’s just been a strain on me.’’ Another one said ‘‘I have a 3-year-old son and it’s really hard. Every time we will tell him not to do something, he does it anyway and then you gotta keep saying it like over and over again, that’s just stressful’’. |
|  | **Daily stress- Emotional Coping Response** Living in stressful daily life situations, women experienced mood swings, feelings of irritability, lack of patience, and emotional distress. A woman said ‘‘I get irritated over everything. I used to be such an easy going mellow you know just carefree person and now it’s like my family irritates me; my friends irritate me. I just want to be left alone; right now like I just don’t talk to me, don’t look at me, like I don’t want to because it’s just like it seems like I was always the person that everybody came to with all of their problems. Hey what can we do with, you know now it’s just I don’t want to hear it. Like take your problems someplace else, I don’t want to hear it’’. Another one said ‘‘My boyfriend told me ‘so what happened to you? You were so cool when we got together and now it’s like I don’t even want to be around you.’ I said well I don’t want to be around you either. Just leave me alone’’. They also felt upset about body shape change. A woman said ‘‘Well I know that I gained quite a bit of weight and I was pretty upset about it. I had gained like 30 pounds by the time I was into my second trimester and I was pretty upset because I was losing weight before I got pregnant and then when I got pregnant I was like can’t keep losing weight, now I have to gain weight. I was really upset about that, even though I really wanted to have children’’. To manage daily stress, women utilized various coping strategies. Some women talked to close family members who would listen to them. A woman said ‘‘Talking definitely helps you eliminate a lot of stress’’. Others tried managing time better, taking a long shower, removing oneself from a problematic situation, doing enjoyable activities, and asking for and accepting help. A woman said ‘‘Make you a schedule, map you out some down time within that schedule so you don’t overwork yourself where you get stressed’’. Another one said ‘‘Take yourself away from the situation’’. On the other hand, some used cigarette smoking to help reduce stress. A woman said ‘‘If you stressed out, you pick up a cigarette cuz that’s first thing you do’’. Many women ate foods for comfort when experiencing stress or negative feelings. A woman said ‘‘I eat more when my kids are stressing me out. I go straight to the kitchen’’. Another one said ‘‘Sometimes you’re just depressed… and you just want to eat, it makes you feel better’’. Many reacted to stress by yelling at their family members or children. A woman said ‘‘I hurt his (son) feelings the other day cuz I yelled at him; he was leaning on me and I was just laying there. You know just snapped on him for no reason’’. Women equivocally said that they would like to talk to their peers about pregnancy experiences. |
| Discussion | Several studies have used focus group discussions to investigate low-income pregnant women’s dietary intake and/or physical activity [45–48]. However, this was the first study to identify key stressors (especially communication with significant others), barriers and motivators for healthy lifestyle practice, and potential drawbacks for monitoring weight among low-income overweight and obese African American and non-Hispanic white pregnant women. These women had unstable and stressful lives and experienced constant negative feelings in response to psychosocial stress. These factors are important because there is increasing evidence that stress has been linked to low birth weight (\2,500 g) and preterm birth (\37 weeks of gestation) [49–51], especially among low-income women [52, 53]. Previous studies reported a negative relationship between pregnant women and their significant others but did not identify the reasons [9, 47]. A recent report showed that pregnant women tended to argue with their partners more than usual [6]. Their frustration mounted as their significant others were too nervous about pregnancy safety and did not understand the pregnant women’s needs. Our previous observations with low-income overweight and obese women suggest that these women tend to worry about less consequential matters or imagine frightening scenarios which have low probabilities of happening. They often think their own experiences are unique; thus, feeling isolated or depressed. They want to learn more about stress management and problem-solving skills to deal with daily psychosocial stressors (e.g., relocation, experience of family tragedy, inability to pay bills, or unemployment) [6]. However, they have minimal access to appropriate resources or are unaware of existing resources. Also, most women prefer not to share daily psychological stress with providers because they are afraid of being judged or think that their providers will not understand them. Previous research showed that low-income women were unlikely to receive adequate advice from health care providers due to providers’ time constraints or providers’ priorities that focus on more acute medical and psychosocial issues [54,55]. Low-income pregnant mothers who experience intense negative feelings are a public health concern because they may not be able to meet their personal health and emotional needs and the needs of their other children. For example, ineffective coping skills will most likely influence their young children’s problem-solving skills in the future. Consistent with previous research [8, 9], women in this study expressed a strong desire to talk to their peers with similar life situations on a regular basis. However, transportation, childcare issues, and absence from work are most likely preventing them from attending face-to-face support groups on a regular basis. One alternative is to provide peer support via group teleconferences. However, our pilot [56] and current intervention studies [57] aimed to help low-income overweight and obese WIC mothers prevent weight gain have consistently shown that more than half of women would not call in at specified times for many reasons, e.g., time conflict, family illness or death, working 2 jobs, relocation, child custody issues, domestic violence, and going through divorce [56]. Therefore, there is an urgent need to have interventions focusing on effective, simple, and practical ways to help low-income overweight and obese pregnant women manage their daily psychosocial stress and negative feelings. Our finding that women withdrew from their social lives is a major concern given the fact that they already experienced lack of social support from their significant others. They also experienced intense stress and negative feelings. |
| Conclusions | In conclusion, the study results have several important implications which are useful to researchers and community organizations as they design weight gain interventions for pregnant women |
| Limitations | not given |
| Recommendations | We recommend that interventions be designed to increase involvement of pregnant women’s significant others and help husbands and boyfriends understand the pregnant women’s needs and concerns. Also, our results show that it is critically important to help these women manage stress and negative feelings. Once they can manage both, they are more likely to be successful in eating healthier and becoming more physically active. However, assessing and addressing psychosocial stress are not included in routine WIC care. Given the fact that nearly 1 million pregnant women receive WIC benefits [65], policy makers may consider inclusion of stress management into routine WIC care to promote better maternal and birth outcomes |

| 10. Title | Rural Pregnant Women's Stressors and Priorities for Stress Reduction |
| --- | --- |
| Publication year | 2012 |
| Author | Bloom et al. |
|  |  |
| **Participant Characteristics** |  |
| Participant group: Pregnant women/mothers (of children up to one year) | Pregnant women |
| Number of children | not given |
| Age | 18-34 |
| Ethnicity | White (91.1) Black (8.7) |
| SES | Low-income rural mothers |
| Recruitment strategy | Flyers were advertised about the study in rural WIC clinics and health departments. |
| Sample size | Twenty-four women were enrolled, and 47 interviews conducted (one participant completed the first interview but could not be reached for the second) (n=23) |
| Eligibility | Eligible participants were pregnant (≤ 32 weeks), low-income, residing in a rural county as defined RURAL PREGNANT WOMEN’S STRESSORS 815 by the Federal Office of Management and Budget (Economic Research Service, 2007), English-speaking, and age 18 or older. |
| **Methods** | The study aimed to describe stress exposures, stress responses, and priorities for stress reduction among a sample of low-income rural pregnant women |
| Theoretical background | A mixed-methods approach, with the qualitative approach predominating |
| Design | A private and safe setting of the woman’s choice, usually her home |
| Study setting | not given |
| Data collection approach | Qualitative interviews |
| Interview topics/focus | 1-1.5 hours |
| interview/focus group duration |  |
| Use of additional resources/prompts | Qualitative interviews were transcribed verbatim and analysed with a qualitative descriptive approach |
| **Findings** | **Women’s Stress Exposures** Scale scores for measures of lifetime exposure to stressors, global perceived stress, and depression and PTSD symptoms are presented in Table 3. Mean Perceived Stress Scale scores were high, and most women described themselves as highly stressed. Financial stress was by far the most predominant stress reported; many struggled to afford basic amenities (e.g., housing, food). This was perceived as particularly stressful when mothers could not provide for their children (e.g., school supplies, birthday parties). For example, Valerie (all names are pseudonyms) was humiliated when other parents paid her son’s way into school events: “I get really frustrated, upset, and mad ... that makes me feel even worse.” Anna Lee had recently served jail time for shoplifting diapers. She said: Because I am telling you, when I can’t get them what they want or need, that—it makes me crazy. That is bad ... I don’t know if I should say this or not, but there’s been times that I will just go ahead and take it, take something ... They just need things and I can’t do it. And it really bothers me badly. Most women had either an unreliable vehicle or none at all, leaving few transportation options other than relying on friends or family and presenting problems for women where, as DeeDee said, “It’s 20 minutes to anything.” Women also commonly complained of high rent and rental housing in poor condition (e.g., gas leaks, broken appliances). For example, Helena’s home was poorly insulated. When she could not afford her high heating bill, her utilities were turned off mid-winter, forcing her to move in with her parents until her parents paid the utility bill. She worried she would be unable to heat the house adequately for a new born in the coming winter. Many women (10 of the 24) were living with extended family in conditions that were often crowded and stressful. For example, Frances lived with her parents and younger sibling, sharing a bedroom with her boyfriend and two children. She described daily disagreements with her mother, adding, “Anything from my mother comes with a pretty hefty price tag.” Nine of the 24 were employed outside the home (primarily in-service industry or caregiver positions). Many reported stressful working conditions, including jobs that were far away or had long hours, low pay, or were only part-time. DeeDee, who drove 45 minutes each way to a minimum-wage waitressing job, said “I get paid every two weeks, and my pay check lasts about a week.” |
| Theme | **Women’s Stress Exposures** Scale scores for measures of lifetime exposure to stressors, global perceived stress, and depression and PTSD symptoms are presented in Table 3. Mean Perceived Stress Scale scores were high, and most women described themselves as highly stressed. Financial stress was by far the most predominant stress reported; many struggled to afford basic amenities (e.g., housing, food). This was perceived as particularly stressful when mothers could not provide for their children (e.g., school supplies, birthday parties). For example, Valerie (all names are pseudonyms) was humiliated when other parents paid her son’s way into school events: “I get really frustrated, upset, and mad ... that makes me feel even worse.” Anna Lee had recently served jail time for shoplifting diapers. She said: Because I am telling you, when I can’t get them what they want or need, that—it makes me crazy. That is bad ... I don’t know if I should say this or not, but there’s been times that I will just go ahead and take it, take something ... They just need things and I can’t do it. And it really bothers me badly. Most women had either an unreliable vehicle or none at all, leaving few transportation options other than relying on friends or family and presenting problems for women where, as DeeDee said, “It’s 20 minutes to anything.” Women also commonly complained of high rent and rental housing in poor condition (e.g., gas leaks, broken appliances). For example, Helena’s home was poorly insulated. When she could not afford her high heating bill, her utilities were turned off mid-winter, forcing her to move in with her parents until her parents paid the utility bill. She worried she would be unable to heat the house adequately for a new born in the coming winter. Many women (10 of the 24) were living with extended family in conditions that were often crowded and stressful. For example, Frances lived with her parents and younger sibling, sharing a bedroom with her boyfriend and two children. She described daily disagreements with her mother, adding, “Anything from my mother comes with a pretty hefty price tag.” Nine of the 24 were employed outside the home (primarily in-service industry or caregiver positions). Many reported stressful working conditions, including jobs that were far away or had long hours, low pay, or were only part-time. DeeDee, who drove 45 minutes each way to a minimum-wage waitressing job, said “I get paid every two weeks, and my pay check lasts about a week.”  **Women’s Stress Responses** Women in this study described feeling chronically stressed; depression and PTSD symptoms were common (Table 2). Nearly 2 out of 3 (n = 14; 61%) participants exceeded the cut score of 16 for clinically significant symptoms of depression, and nearly 1 in 4 reported moderate to severe levels of PTSD symptoms. Women reported that substance abuse was highly prevalent in rural communities, and often attributed this to boredom, depression, and “nothing to do.” Anna Lee lamented, “I am telling you; we sit in the house a lot and don’t do anything at all.” Frances described how unstructured time and boredom increased her symptoms of depression saying, “time does nothing but hurt me.” Because of state mandatory reporting requirements, we did not directly ask these pregnant women about their own use of drugs or alcohol. Formal resources (health care systems, police, women’s shelters, welfare, and other services) were seen as limited in their ability to help and often too far away. Some women also felt judged, poorly treated, or gossiped about in-service settings. Extended families were often highly interdependent, providing financial help, housing, childcare, and transportation to work, school, and appointments. However, some families had longstanding dysfunction and were sources of stress and conflict. Many women valued being “strong,” and interdependence could also chafe. Patricia said, “I don’t think it’s healthy to always be around family that much. I love ‘em to death, but we’re trying to have our own family too, you know?” In addition to relying on interpersonal networks, women employed multiple strategies to manage stress. A few women relied on religious faith; many reported they coped by “just being strong and ... keep on going;” trying to ignore bad feelings and “just get over it;” and staying occupied sleeping, watching TV, web-surfing, or cleaning house. Several women focused energy on their children. Tia said, “Every day just having them, watching them, seeing them, spending that time with them takes a lot of it away.” Many women found talking about stress helpful. As Bree Anne said: I have to talk about it. ‘Cause if I don’t say nothin’ about it, I’m just going to let it sit here and let it all build up ... Being stressed ... as long as you have someone to talk to, it’s not as hard to cope with. However, talking about stress could represent a threat to privacy, and thus some women avoided it. A common theme was that in a rural community, “everyone knows everyone” and gossip was a constant problem. Bree Anne said, “They keep your business in their business, whether they know you or not.” Some had been badly betrayed by a trusted confidante. As Irene said, “If you tell somebody something the whole town is going to hear RURAL PREGNANT WOMEN’S STRESSORS 817 about it ... it just makes me feel hurt.” Many women reported having few or no friends and feeling isolated and lonely. Some women had acquired “bad reputations” that further increased their isolation and were difficult to shake. Crystal said, “People still think I’m still that person.” Even family members ‘reputations could make life difficult for women like Anna Lee, who contemplated marrying a violent partner in part because “my last name kills me in this town.” |
|  |  |
|  |  |
|  |  |
|  | **Women’s Stress Responses** Women in this study described feeling chronically stressed; depression and PTSD symptoms were common (Table 2). Nearly 2 out of 3 (n = 14; 61%) participants exceeded the cut score of 16 for clinically significant symptoms of depression, and nearly 1 in 4 reported moderate to severe levels of PTSD symptoms. Women reported that substance abuse was highly prevalent in rural communities, and often attributed this to boredom, depression, and “nothing to do.” Anna Lee lamented, “I am telling you; we sit in the house a lot and don’t do anything at all.” Frances described how unstructured time and boredom increased her symptoms of depression saying, “time does nothing but hurt me.” Because of state mandatory reporting requirements, we did not directly ask these pregnant women about their own use of drugs or alcohol. Formal resources (health care systems, police, women’s shelters, welfare, and other services) were seen as limited in their ability to help and often too far away. Some women also felt judged, poorly treated, or gossiped about in-service settings. Extended families were often highly interdependent, providing financial help, housing, childcare, and transportation to work, school, and appointments. However, some families had longstanding dysfunction and were sources of stress and conflict. Many women valued being “strong,” and interdependence could also chafe. Patricia said, “I don’t think it’s healthy to always be around family that much. I love ‘em to death, but we’re trying to have our own family too, you know?” In addition to relying on interpersonal networks, women employed multiple strategies to manage stress. A few women relied on religious faith; many reported they coped by “just being strong and ... keep on going;” trying to ignore bad feelings and “just get over it;” and staying occupied sleeping, watching TV, web-surfing, or cleaning house. Several women focused energy on their children. Tia said, “Every day just having them, watching them, seeing them, spending that time with them takes a lot of it away.” Many women found talking about stress helpful. As Bree Anne said: I have to talk about it. ‘Cause if I don’t say nothin’ about it, I’m just going to let it sit here and let it all build up ... Being stressed ... as long as you have someone to talk to, it’s not as hard to cope with. However, talking about stress could represent a threat to privacy, and thus some women avoided it. A common theme was that in a rural community, “everyone knows everyone” and gossip was a constant problem. Bree Anne said, “They keep your business in their business, whether they know you or not.” Some had been badly betrayed by a trusted confidante. As Irene said, “If you tell somebody something the whole town is going to hear RURAL PREGNANT WOMEN’S STRESSORS 817 about it ... it just makes me feel hurt.” Many women reported having few or no friends and feeling isolated and lonely. Some women had acquired “bad reputations” that further increased their isolation and were difficult to shake. Crystal said, “People still think I’m still that person.” Even family members ‘reputations could make life difficult for women like Anna Lee, who contemplated marrying a violent partner in part because “my last name kills me in this town.”  **Women’s Priorities for Stress Reduction** When asked what would help reduce stress for rural women, participants suggested connecting people socially through community centres, providing mentoring, and expanding the availability of formal resources (utility help, options for juvenile offenders, housing assistance). However, the most common response was a “good job,” providing a good wage, benefits, and flexibility around children’s needs. Some also defined a “good job” as work that was meaningful, making a difference or helping others. Women believed a good job would decrease their financial stress, relieve boredom and loneliness, provide a sense of purpose and of making a difference in the world, increase self-esteem, and decrease dependence on extended family and others. Many saw higher education or vocational training as the key to a good job, but relatively few had attempted it. Many had struggled to complete high school. Local options were limited; women saw financing of education or job training, transportation, childcare and the need to work around employment, family, and other responsibilities as barriers. This was particularly challenging for women with poor support. For example, Crystal grew up in foster care and had no family. She began college, but quit when she became pregnant, saying, “I can’t go to class with a baby on my hip. “Despite the barriers, women envisioned other benefits to education or job training. For example, the opportunity to make friends and decrease her isolation appealed to Melissa, who said, “You could meet your best friend. I think it would be a big advantage.” |
|  |  |
|  | A common mental image of rural America is of peaceful, pastoral settings and idyllic, sheltered existences (Gale, 2010). However, the rural pregnant women in this study disclosed high levels of overall stress, with financial stress and a lack of access to transportation, affordable housing, and employment options reported as significant stressors. Nearly two out of three women in this study reported current symptoms consistent with major depression, and nearly one in four reported moderate to severe levels of current PTSD symptoms. A limitation of this predominantly qualitative study is that this sample of women, a majority of whom described themselves as “highly stressed,” was small and self-selecting. However, our findings are consistent with other research reports of high prevalence of stress and depressive symptoms among low-income rural women (Bhandari et al., 2008; Hillemeier et al., 2008; Jesse & Swanson, 2007; Probst et al., 2005). It is also consistent with research that has found maternal stress is significantly interrelated with maternal mental health symptoms (Dailey, et al., 2011; Jesse & Swanson, 2007; Kendall-Tackett,2007; Koleva et al., 2011; Seng et al., 2011). We note that the women in this study also had high PTSD symptomatology and reported substantial lifetime violence exposure; one in five were in abusive relationships at the time of the interview. Yet participants rarely discussed violence as a stressor in qualitative interviews. It is possible that these rural women did not feel comfortable or safe discussing violence at length in the qualitative interviews. Given their significant financial distress and lack of resources, it is also possible that some of the pregnant women in this study who were living with violence may not have seen it as their most pressing or immediate problem, as other studies have found (Curry, Durham, Bullock, Bloom, & Davis, 2006; Libbus et al., 2006). Lindhorst et al. (2005) explain women’s responses to violence in a stress and coping model, suggesting women make sense of violence by evaluating the seriousness of the situation and its threat of harm to themselves and their children. This evaluation takes place in the context of both their current situation—what’s happening and how bad it seems—and their prior experiences with violence. Their responses to the violence are also shaped by their goals, commitments, and beliefs, as well as the resources and options they see as available to them. We suggest rural clinicians should screen for violence exposures and connect women with formal resources so they can make safety plans based on their level of danger, their priorities, and their resources (Davies, Lyon, & Monti-Catania, 1998). We also suggest rural clinicians remain mindful that a pregnant abused woman is making decisions in a context that is shaped by many factors and needs support in the decisions she makes. |
| Limitations | We therefore strongly recommend that clinicians and researchers seeking to decrease maternal stress and improve rural maternal-child health outcomes consider empowering these women, using collaborative, community-partnered approaches that include rural, low-income mothers’ voices in conceptualization, design, implementation, and evaluation of the results |
| Recommendations | Not given |

| 11. Title | Development of a Psychological Intervention to Address Anxiety During Pregnancy in a Low-Income Country |
| --- | --- |
| Publication year | 2020 |
| Author | Atif et al. |
| **Participant Characteristics** |  |
| Participant group: Pregnant women/mothers (of children up to one year) | Pregnant women |
| Number of children | not given |
| Age | The mean age of the women was 26 years |
| Ethnicity | not given |
| SES | Low-income urban, peri-urban, and rural populations |
| Recruitment strategy | Trained research assistants approached consecutive pregnant women as they attended an obstetrics department |
| Sample size | 19 women with perinatal anxiety participated in the study |
| Eligibility | Not given |
| **Methods** |  |
| Study aim | The study aimed to develop a culturally appropriate, feasible, and acceptable psychological intervention for perinatal anxiety in the context of a low-income population in Pakistan. |
| Design | A mixture of research methods |
| Study setting | The study was conducted in the Obstetrics Department of the Holy Family Hospital, Rawalpindi, a public hospital with a catchment population of over 7 million drawn from urban as well as peri-urban and rural areas of the district. |
| Definition/conceptualisation of stress and/or anxiety | not given |
| Data collection approach | We collected data through in-depth interviews with pregnant women attending the outpatient clinics |
| Interview topics/focus | not given |
| interview/focus group duration | not given |
| Use of additional resources/prompts |  |
| Analysis | All interviews were recorded, transcribed verbatim, and analysed using the framework analysis. The framework analysis allows the data to be analysed systematically in five stages: familiarization, development of the thematic framework or index, indexing, charting, and interpreting the data |
| Rigour (Saturation, reflexivity) | not given |
| Theme | **Sources of Anxiety During Pregnancy**- A strong theme emerging from interviews with the mothers was traumatic experiences related to previous pregnancies. These included unplanned or unwanted pregnancy, medical complications related to pregnancy, preterm delivery, and loss of or health problems in the child. I am very tense; it’s my 6th pregnancy, and I only had two children alive. My two sons died after 15 and 22 days of birth and one daughter died during pregnancy (miscarriage) (IDI-mother 10). A majority of the mothers reported lack of trust in the health services and fear about losing their own or their baby’s life during labour/delivery. Most women, unable to afford private treatment, sought care from the public hospital and often struggled to pay for any extra medical tests or medicines prescribed by their doctors. When I think about delivery and recall the comments of other patients regarding hospital services, delivery and operation, it scares me. What if I will lose my baby or if I will die? (IDI-mother 11). The relatively poor quality of care available in public sector hospitals was also frequently mentioned by health professionals, who attributed it to lack of funding and overburdened health systems. Another theme that emerged was about the lack of support from husbands or other family members during pregnancy. Many women described not having anyone to talk to about their concerns. This account was supported by the health professionals responsible for their care during delivery. The behaviour of their in laws and husband is often not good towards them; they don’t talk nicely to them, don’t bring them for check-ups, don’t take their problems seriously. Of course, all these things have an impact on patients (IDI-gynaecologist). Another source of anxiety for the women, especially those who already have daughters, was pressure from their husbands and in-laws to give birth to a male offspring. The worry during pregnancy is, mostly thinking about (whether it is going to be) a son or a daughter. The people around you are discussing this issue again and again. That is why women get upset. When the mind is upset all the body gets upset (IDI-mother).  **Manifestations and Impact of Anxiety During Pregnancy**- A key theme in this area was the range of somatic symptoms experienced by women, including feeling that their “heart was sinking,” palpitations, breathlessness, sweating, dizziness, restlessness, weakness, feeling drained, trembling, numbness, aches, and pains. Women largely attributed these symptoms to a physical cause, which led to concerns about their own and the baby’s well-being. When I am anxious, my body starts shivering, Blood pressure gets high. I have a headache and can’t sleep (IDI-mother 14). The main emotional manifestations included feeling worried, fearful, apprehensive, mentally disturbed, tense, angry, withdrawn, uneasy, or tearful. Different crazy thoughts come in my mind. I feel so fearful not knowing what will happen to me or my baby (IDI-mother 17). With respect to the impact of anxiety, the main themes that emerged were related to the women’s personal well-being, their relationship with people around them, and their relationship with their children. An obstetrician with over 25 years of work experience stated: When any woman is psychologically disturbed, her wellbeing gets affected, her diet gets affected, health is also affected. Sometimes her labor also gets effected, as her pain threshold gets lower and the labor can be prolonged, and also the post-delivery recovery takes longer. Most of time she keeps lying in bed, won’t be able to feed her child properly because of lack of support |
|  |  |
|  |  |
|  |  |
|  | **Protective Factors for Anxiety During Pregnancy**- Many of the health professionals and the women reported feeling that availability of adequate antenatal care and an empathetic attitude from health professionals could help ease the expectant women ’s anxiety and motivate them to attend antenatal check-ups. If we give individual attention to the patient and give some extra time to them, their anxiety could be treated. Instead of medicines, we need to talk to them and give some counselling to them (IDI-gynaecologist 4). Another strategy that appeared to protect some women from anxiety was turning to faith, using prayers and religious rituals to deal with their symptoms. All I do is to separate myself from everyone and offer my prayers to God. This makes me stay relaxed and calm (IDI-mother 9). In terms of protective factors, support from family and friends emerged as a strong theme. This included both direct personal support and support from a distance through social media or mobile phones. Women described that talking to family and friends, in person or through social media, and spending quality time with their children helped them cope with their symptoms of anxiety. |
|  | **Desired Features of a “Talking Therapy” for Anxiety**- A majority of the women (18/19) agreed on the potential for a talking therapy to help with their anxiety. They felt that someone who listened to their problems, tried to understand them, and provided suggestions that could make them feel better would be very welcome and that they would find time to engage with such a person. A majority of the participants expressed a need for an intervention which was relevant to their everyday lives and day to-day problems, helped with their well-being, and instilled hope. It will be beneficial, it will increase awareness and give women an opportunity to share their concerns, which could help them to offload (IDI-mother 7). A strong theme that emerged in this area was the preference for someone who was empathetic, caring, and courteous, irrespective of whether they were health professionals or not. There was general agreement that such a person should be female and properly trained and supervised. It is not important that the person is doctor, midwife on anything; what matters is that the person is a woman, who can listen to other women and who is kind-hearted. I think it is better to train women and then deliver the programme through them (IDI-mother 10). There were conflicting views among the women regarding the preferred format of the sessions. Some suggested that a group setting for intervention delivery would be optimal, as it would provide an opportunity to meet and share their problems with other women, while others felt that individual sessions are preferable, as problems could be shared confidentially without feeling embarrassed in front of others. With regard to session timings, duration, and venue, it was suggested that sessions should be held during the morning, should not be more than an hour, and should be held at the hospital or another health facility. Delivering the talking therapy at the same visit as when the woman attended her routine antenatal appointments was suggested as a way to improve attendance. hospitals are better as women already come here. It will not be possible to receive intervention at homes as there is no privacy there (IDI-mother 16). A majority of the women were poor and dependent on their family members for travelling to the hospital. |
| Discussion | We have described a psychosocial intervention developed specifically for women with perinatal anxiety in low-income countries. The intervention development process adhered to the MRC (UK) framework for development and evaluation of complex interventions (33). It was informed by the evidence base for what works in perinatal anxiety (21, 22), including: a. A well-established theory underpinning the process of expected change b. Detailed formative research to contextualize the intervention to the target population c. Use of evidence-based elements and strategies from existing well-established approaches. Preliminary feedback from participants indicated that the intervention was acceptable, feasible, and perceived to be helpful by the women receiving it. Perinatal anxiety is a critical public health priority, given the high prevalence, its tendency to predispose women to subsequent postpartum depression, and the negative impacts on child development and health outcomes. Lack of trained mental health specialists to deliver psychosocial interventions is a major barrier to addressing this problem (45). This simple intervention can be delivered by non-specialists under supervision and thus, if shown to be effective in subsequent large-scale evaluations, could address the treatment gap for common mental disorders in LMICs (46). Similar approaches have demonstrated clinical benefit and utility in high-income settings. For example, an evaluation of the UK’s Improving Access to Psychological Therapies program (IAPT) (47) found a substantial reduction in depression and anxiety in people who attended low-intensity interventions delivered by university graduates with a few months of training. A meta-analysis reviewed similar low-intensity interventions and found these approaches to be effective even for individuals with symptoms of severe depression (48). It should be emphasized, however, that while such so-called “low-intensity” interventions could play an important role in reducing the treatment gap for common mental disorder in LMICs, they are only one component of a collaborative stepped care model of service delivery. This intervention was designed to address symptoms of anxiety before these become chronic, severe, and debilitating, necessitating more specialized care in the stepped-care pathway. Such interventions can therefore play an important role in prevention, allowing women to learn strategies for stress management and problem solving before the symptoms become ingrained. This model of care fits well with the staging approach to identification and management of mental disorders advocated by the Lancet Commission on Global Mental Health (49). Women who do not respond to this level of care or who go on to develop more serious symptoms should be referred to and able to access more specialized care. We limited our study population to women living within a 20 km radius of the General Hospital. While our purposive sampling included women from a range of ages, neighbourhoods, and socioeconomic gradients, generalization of our findings to other populations should be done with caution. However, our key findings with regards to the sources, manifestations, and impact of anxiety were remarkably similar to existing literature from a range of different settings and contexts (3–11). In matching our intervention approach with available “psychosocial” interventions, we limited ourselves to the WHO mhGAP-IG. We recognize that the intervention packages within the mhGAP-IG are not specifically designed for anxiety, but are described as “transdiagnostic,” given the overlap between management of depression, anxiety, and other stress-related conditions. We are cognizant that other approaches, such as acceptance–commitment and mindfulness therapies, not covered by the mhGAP-IG, might also contain elements that could potentially be useful for our intervention. Neither did we consider anxiety-specific protocols from HICs, given that matching these to the population and health-system context would be problematic. |
| Conclusions | This new psychosocial intervention for perinatal anxiety, based on principles of CBT, was found to be acceptable and perceived to be helpful by the women who received it. The intervention was feasibly delivered by non-specialist professionals after brief classroom training and a few practice sessions under supervision. It therefore has the potential to address this important but neglected condition in LMICs. However, effectiveness studies are required prior to recommendations for its integration into perinatal care. |
| Limitations | not given |
| Recommendations | not given |

| 12. Title | The role of fear of childbirth in pregnancy related anxiety in Iranian women: a qualitative research |
| --- | --- |
| Publication year | 2017 |
| Author | Arfaie |
| **Participant Characteristics** |  |
| Participant group: Pregnant women/mothers (of children up to one year) | Pregnant women |
| Number of children | not given |
| Age | 18-41 |
| Ethnicity | Different ethnic groups |
| SES | Different SES groups |
| Recruitment strategy | Mothers were recruited through healthcare centres in Tehran |
| Sample size | Twenty-eight women with a history of anxiety and fear of childbirth participated in the study |
| Eligibility | Having spouse and singleton pregnancy, mothers who had not experienced any kind of mood disorders in themselves or their blood relatives, lack of burdensome accidents during the past 6 months, lack of chronic diseases such as cardiac diseases, thyroid, diabetes and adrenal disease, their current pregnancy was not classified as high-risk gestation and those who preferred caesarean -section. |
| **Methods** |  |
| Study aim | This study aimed to explore components and dimensions of pregnancy anxiety. |
| Design | A qualitative study was collected through individual in-depth semi-structured interviews with 28 women who were selected using purposive sampling. |
| Study setting | The interviews were conducted in a quiet environment |
| Data collection approach | Data was collected through individual in-depth semi-structured interviews with 28 women who were selected using purposive sampling. |
| Interview topics/focus | The interviews began with a general question: “How do you feel about childbirth?” and gradually progressed to specific negative experiences and fears about childbirth based on the interview guide. |
| Analysis | Data analysis was conducted using MAXQDA software. |
| Rigour (Saturation, reflexivity) | not addressed |
| Theme | **Childbirth process-** This category was the first category in this research. It is clear that pregnant women consider childbirth to be a painful, prolonged and overwhelming experience which requires an excessive amount of power, energy, and support. They can unequivocally conclude that normal delivery is troublesome and they lack sufficient self-confidence to tackle it. For instance, a master student who was a 29-year-old mother commented accordingly “from my standpoint, delivery means pain, fear and fear of pain.” And an employee who was a 29-year-old woman said “vaginal delivery could be very painful, and prolonged, so I prefer operational delivery. I have been thinking about this problem since the early days of my pregnancy but now I think that I am scared of delivery and tell myself I cannot have any control and tackle it, I do not have enough strength to do that, I may die during normal delivery and cannot pass through it.” Another important problem from mothers’ viewpoints was the need for support and avoidance of loneliness. A 28-year-old female engineer who was pregnant believed that “loneliness during labor is very stressful.” And she continued “I am always afraid that I will be on my own during my labor and at my delivery time that my mother or my husband will leave me alone. Fear of incompetency and the need for emergency caesarean section were among other problems related to normal delivery. A 22-year-old housewife said “I ask myself that what will happen if I tolerate its severe pain and cannot give birth? Can I have normal delivery? |
|  |  |
|  |  |
|  |  |
|  | **Childbirth time-** Childbirth time along with its concerns and fears was another main category in this study. A pregnant woman always asks herself “what time would my delivery be? Do I get to hospital on time? What happens if preterm labor treats me?” We see such doubt in their stories about childbirth time. A 32-year-old housewife said in this regard “I am afraid of preterm labor because I have experienced that before, when I was admitted to hospital. I am afraid that it may happen again and may the health of the child I am carrying will be at risk. Fear of unknown delivery time was another notable problem in this study so that some mothers had engaged their minds with it. A faculty member, a 39-year-old woman told us, “The time of vaginal delivery is not clear. It may take place at any time, night or morning, you don’t know the exact time, or who will be there to help you”. Fear of late arrival to hospital was another anxiety-provoking factor for pregnant mothers so much so, that they mentioned it repeatedly in their interviews. A 28-year-old woman who was an engineer sadly stated, “I am permanently anxious and ask myself what will happen if I don’t arrive at hospital on time? What may occur if arrive late and my amniotic sac ruptures, or my husband is not at home, or he arrives late. No one will be at home to take me to hospital.” |
|  | **Childbirth complications**- The third category in this study was about possible severe consequences of normal delivery. Mothers are always anxious about neonate’s injuries and their well-being after childbirth. So they may keep thinking about delivery risks. This finding was explored in our study as well as other works conducted by other researchers. Fear of bleeding and fatality were among such consequences. A 24-year-old housewife with a prior history of postpartum bleeding said, “I experienced severe bleeding in my last delivery and my chest was painful. I am afraid it may happen again. I think about “what will happen if I go to hospital and never come back?” I keep thinking about this constantly. Mood changes and postpartum depression were other areas of fear. A 22-year-old housewife during her 36th week of pregnancy, whose relatives had experienced this disorder said “I am afraid of changes in my mood after delivery, and afraid of getting depressed, because I have seen some of my relatives that had post-partum depression and preferred to be alone.” Possible delivery accidents and injuries, particularly genitalia injuries, were frequently noted by mothers in this study. A 35-year-old housewife who was overweight said “I am afraid that terrible accidents could take place during my labor. Unfavourable stories that have happened to others have affected me. I have seen some people who have experienced vaginal delivery and have had genitalia prolapse and vaginal dilatation. Then, I ask myself “what can I do if these problems happen to me? Because of these problems, caesarean is better. I have heard some women saying that they have had many problems in their sexual functions. They said that normal delivery was not satisfactory.” Fetal health problems and fear of its injuries during labor was considered as one of the most important and perhaps first ranking of fears from the standpoint of some mothers. A 29-year-old pregnant woman stated “Sometimes, I think that they cannot deliver my baby by normal delivery without any problems, so what will happen if my baby is hurt during delivery.” |
| Discussion | It is believed that the information about experiences of mothers in their pregnancy and how it affects their psychological health is not enough or acceptable. So, it is important to explore local evidence regarding women’s psychological problems in pregnancy. With respect to the findings of our research, it is clear that a mothers’ anxiety arises from a sense of uncertainty about the forthcoming process of pregnancy and delivery h and they are not sure if they will go through the process of giving birth without any complications (23). This uncertainty may originate from the risks associated with pregnancy care, support and childbirth process management. Therefore, fear of childbirth was focused on four categories in our study: Fear of delivery process, delivery time and delivery complications and healthcare related fear. Rouhe (13) categorized fear of childbirth into fear of pain, social background, prior negative childbirth experience, history of abuse and violence, horror stories, personality, social support and mental health problems. 4.1. **Fear of childbirth process** It was the first extracted category in our study to which had been referred to in many studies (24, 30). Nilsson (24) in her paper titled “lived experience of childbirth in women with severe fear of childbirth” found that in nulliparous cases, women feel uncertain of their ability to bear and give birth to a child and in multiparous cases, they described their experiences of suffering in relation to the care they received during childbirth. This mainly concerned pain and negative experiences with staff. Salari (25) in her paper as “stressful factors in pregnancy” showed that fear of pain, being left alone during delivery, feeling of incompetency and need for emergency caesarean were the most important factors of a stressful pregnancy. Theresa, (12) in his study, demonstrated that fear of childbirth was observed among 16 % (27/169) of women with poor social support, 33 % (24/73) of women with combined anxiety and depression, and 28 % (20/72) of women with a previous negative overall birth experience which were all followed by poor social support. Also, giving birth for the first time and a high educational level were associated with fear of childbirth. Faisal (26) believed that labor pain and fear of one’s own incompetency (65%) were some of the important factors of fear of childbirth. Findings of Abbaspoor (27), Khorsandi (28), Sjorgen (29) and Szeverenyi (30) were also consistent with our findings. Accordingly, we can conclude that fear of delivery process is mainly dependent on fear of pain, thinking about inability to give birth and request for emergency caesarean –section. Otherwise, in all cases, mothers had low self-confidence for normal delivery. Obviously, development of painless delivery methods in all maternity wards and hospitals, and acquainting women with their potential for normal delivery play critical roles in controlling fear and anxiety in pregnant women. **4.2. Time of childbirth** This was the second category in this study. Although EDC is estimated in the majority of pregnancies, a great deal of deliveries don’t occur at a certain time and sometimes, preterm labor happens. Another problem is that delivery may take place at night, in the evening or on holidays. Therefore, the exact time of delivery is not clear for mothers. Consequently, in our study pregnant women were concerned about this problem. This finding was consistent with that of Salari (25) regarding pregnancy stressors. She pointed out that preterm labor and fear of late arrival to hospital are among prominent factors that concern mothers about normal delivery. Sereshti (31) in her study concluded that lack of access to a physician or to suitable healthcare on holidays were concerns about prenatal services for pregnant mothers. As a result, it is clear that in many cases fear of delivery time is due to uncertainty about receiving care or services at any time and lack of support and assistance whenever necessary. It seems that reassuring pregnant women in this field is necessary and we should point out that a husband’s help and involvement has a unique role in decreasing a mothers’ anxiety. **4.3. Childbirth complications** The third category in this study was concern with delivery and its unpredictable consequences, which was classified into five subcategories including: fear of bleeding, fear of death, postpartum depression, delivery accidents, genitalia injuries and fetal health problems. In fact, delivery is a unique physiological experience during the women’s journey of life, however, sometimes adverse consequences are inevitable that can be seen in the stories told by women. Injury to the mother or her neonate and complications after vaginal delivery such as vaginal prolapse, urinary incontinence and sexual dysfunction were the main findings in the studies conducted by others (23, 25, 28). Khorsandi (29) in his study wrote that fear of inflicting injuries on the neonate and causing defects in the baby were the most important factors for selective caesarean demand. Szeverenyi (31) reconfirmed it and expressed “definite assurance of maternal and child health is one of the most important pillars of labor and delivery, and if the mother cannot ensure sufficient trust and confidence in this field, the outbreak of fear and anxiety for her would be inevitable. **4.4. Healthcare quality** It is clear that how mothers feel about safe delivery, and their well-being completely depends on the gynaecologist, staff and the environment of the hospital. We can see pregnant women’s respective concerns in their statements. Fasial (26) believed that anxiety about the delivery was related to lack of trust in the obstetrical staff (73%), maternity ward staff’s relationship with mothers and the level of hospital. In Salari’s (25) study, fear of hospital facilities and staff and their contact was notable. Khorsandi (29) in her study, found that fear of hospital environment, healthcare quality, injection and loneliness were the main factors of childbirth fears. Sjorgen (30) believed that mothers do not have enough trust in maternity staff and their interventions. Sereshi (31) found mothers’ perception of quality of healthcare services in their study. She stated that many participants were dissatisfied with the care provided in the hospital during pregnancy and afterwards for the following reasons: being left alone, not being referred to centres with better equipment, no follow-up visit, and lack of appropriate action in emergency cases. She continued ‘lack of understanding of the mother’s situation and poor communication of the treatment team at the obstetrics and gynaecology departments worsened the mothers’ problems and intensified their distress. So it is clear that maternity staff should be re-educated on observance of medical ethics and professional rules in their practices and change their attitudes and behaviour towards clients to reduce pregnant women’s fear and anxiety all over the world. Generally, as fear of childbirth includes negative attitudes toward labor and increases the risk of prolonged labor, the need for pain relievers (16), dystocia, PTSD (11), postpartum depression, mother and neonate bonding problems and request for selective CS (29), early assessment of mothers, particularly their fears and concerns, is necessary. |
| Conclusions | The findings of this study highlighted that the main causes of maternal anxiety about childbirth are due to fear of damage to mother and baby and lack of trust to the quality of maternity care and staff commitment. Consequently, concerning the results of this study, it is necessary to revise prenatal care services in order to develop prenatal education, support and coping strategies, and to enhance women’s knowledge and confidence about childbirth to reduce their pregnancy anxiety and fear of childbirth |

| 13. Title | What are women stressed about after birth? |
| --- | --- |
| Publication year | 2019 |
| Author | Ayers et al. |
| **Participant Characteristics** |  |
| Participant group: Pregnant women/mothers (of children up to one year) | Women 6‐12 weeks after birth |
| Number of children | not given |
| Age | Women's age ranged from 21 to 42 |
| Ethnicity | The majority were White European (94.3%), followed by Asian (2.8%), other (1.9%), and African (0.9%) |
| SES | Many women were educated to degree level (43.2%), and only two women had no educational qualifications (1.4%). Most were in employment (83%) and in professional occupations (53%) |
| Recruitment strategy | Recruitment took place in 14 NHS hospitals in England from November 2013 to December 2014 |
| Sample size | 148 women consented to participate |
| Eligibility | Women aged 18 years or older and had given birth to a live infant after 26 weeks’ gestation. |
|  |  |
| **Methods** |  |
| Study aim | The current study aimed to address these methodological issues by identifying key stressors during the perinatal period, using a method that encourages open and honest reporting. |
| Design | A qualitative study design |
| Study setting | NHS hospitals |
| Data collection approach | Women wrote anonymously about a situation they found stressful as part of the Health after Birth Trial (HABiT) of expressive writing. Transcripts were analysed for categories of stressors and cross‐cutting themes. |
| Interview topics/focus | Stressors |
| Analysis | Content analysis |
| Theme | **Problems with the baby's health** Forty‐eight women (32.4%) wrote about problems with their baby's health. This included digestive problems, long‐ term impact, serious health problems, and experiences on the neonatal intensive care unit (NICU). The most common subcategory was digestive problems where 12.2% of women reported struggling to cope with their babies’ feeding or digestive problems and how this led to  feelings of helplessness. My 8-week-old daughter has colic and has had this for 4 weeks now. For hours she squeals on and off… it takes patience with myself and with her also. I find it hard not to get irritable and not to shout… I know this is not her fault and that she needs calm and comfort but I feel useless. Acute health concerns and feelings of fear, terror, and helplessness were described by 7.4% of women in situations such as their baby having a very high fever or vomiting blood. I picked her up and she was boiling hot… she kept being sick and when I checked her temp it was 38.8. I told [partner] we had to go to A&E now… I was so scared she had meningitis or was going to have a fit and then be brain damaged…I finally got her settled at 5.30 am then I got in my bed next to her and cried because I was scared for her and scared that I couldn't cope being a mum and I wanted my mum. The long‐term impact of such health issues was a stressor for 6.1% of women who were anxious about their baby being severely ill again. …since then I have been worried about it happening again, so much so I couldn't sleep for the first few days after it happened and dreaded putting her down in her cot in case it happened again. Women whose babies had congenital abnormalities also described worrying about the long‐term effect on the baby. Women (4.7%) whose babies had been on NICU reported this as stressful, affecting their feelings about themselves, and making it harder to bond and care for their baby. …he was hooked up to monitors, had wires and tubes all over him, and a mask on to make sure he was breathing okay. I didn't know what he looked like, what colour hair he had, what his eyes were like… I felt useless too. I couldn’t just pick him up and hold him and look after him like a normal new parent.  **Breastfeeding** Thirty‐five women (23.7%) reported breastfeeding stressors of feeling pressured by others to breastfeed, feeling like a “bad mum” for not wanting to breastfeed, or wanting to breastfeed and not being able to. Pressure to breastfeed was reported by 15.5% of women who wrote of finding breastfeeding “agony,” and being in “constant pain.” Women reported feeling anxious, guilty, and desperate to give up breastfeeding but feeling like they had to continue. It seems like it’s sold be the most natural thing in the world but it is hard work…There are so many breastfeeding Nazis out there who want to make you feel bad for bottle feeding, or even thinking about it, that no wonder many women, me included, feel anxious and guilty about how we feed our children. Another 5.4% of women wrote about feeling like a bad mum for not wanting to breastfeed, that they were letting their baby down and other people would think they were a bad mother. I gave up at 6 weeks and started bottle feeding whilst expressing milk until my supply dwindled at 11 weeks. At the time I felt so guilty to have let [my baby] down… I still feel I have to justify bottle feeding. Everyone has to hear my ‘whole story’ as to why I’m a terrible mother who bottle feeds. Similarly, 2.7% of women reported wanting to breastfeed but not being able to and feeling upset and/or that they had failed. The breastfeeding really wasn’t working… I had no choice but to give up on breastfeeding and |
|  |  |
|  |  |
|  |  |
