# Supplementary material for: Women’s experiences and perceptions of anxiety and stress during the perinatal period: a systematic review and qualitative evidence synthesis
Source: BMC Pregnancy Childbirth. 2021 Dec 6;21:811. doi: 10.1186/s12884-021-04271-w (PMC8647378; doi:10.1186/s12884-021-04271-w)
Supplement: Supplementary file 2 — Additional file 2:. [file 12884_2021_4271_MOESM2_ESM.docx]

| Study | Was there a clear statement of the aims of the research? | Is a qualitative methodology appropriate? | Was the research design appropriate to address the aims of the research? | Was the recruitment strategy appropriate to the aims of the research? | Was the data collected in a way that addressed the research issue? | Has the relationship between researcher and participants been adequately considered? | Have ethical issues been taken into consideration? | Was the data analysis sufficiently rigorous? | Is there a clear statement of findings? | How valuable is the research? | Overall assessment |
| --- | --- | --- | --- | --- | --- | --- | --- | --- | --- | --- | --- |
| Affonso | yes | yes | yes | can't tell | yes | no | can't tell | can't tell | yes | yes | Minor concerns |
| Bloom | yes | yes | yes | yes | yes | can't tell | can't tell | can't tell | yes | yes | Minor concerns |
| Copeland | yes | yes | yes | yes | yes | can't tell | yes | yes | yes | yes | Minor concerns |
| Evans | yes | yes | yes | yes | yes | can't tell | yes | can't tell | yes | yes | Minor concerns |
| Rosario | yes | yes | yes | yes | yes | can't tell | yes | yes | yes | yes | Minor concerns |
| Arfaie | yes | yes | yes | can't tell | yes | can't tell | yes | yes | yes | yes | Minor concerns |
| Ayers | yes | yes | yes | yes | yes | can't tell | can't tell | can't tell | yes | yes | Minor concerns |
| Chang | yes | yes | yes | yes | yes | can't tell | can't tell | can't tell | yes | yes | Minor concerns |
| Atif | yes | yes | yes | can't tell | yes | can't tell | yes | can't tell | yes | yes | Minor concerns |
| Harrison | yes | yes | yes | yes | yes | yes | can't tell | yes | yes | yes | No concerns |
| Rowe | yes | yes | yes | yes | yes | yes | can't tell | can't tell | yes | yes | Minor concerns |
| Razurel | yes | yes | yes | can't tell | yes | can't tell | can't tell | can't tell | yes | yes | Minor concerns |
| Stevenson | yes | yes | yes | can't tell | yes | can't tell | can't tell | can't tell | yes | yes | Minor concerns |

**Supplementary File 2: Assessment of Methodological Limitations**
