# Supplementary material for: Women’s experiences and perceptions of anxiety and stress during the perinatal period: a systematic review and qualitative evidence synthesis
Source: BMC Pregnancy Childbirth. 2021 Dec 6;21:811. doi: 10.1186/s12884-021-04271-w (PMC8647378; doi:10.1186/s12884-021-04271-w)
Supplement: Supplementary file 3 — Additional file 3:. [file 12884_2021_4271_MOESM3_ESM.docx]

**Supplementary File 3. Summary of Review findings**

| **Social Support: Summary of review finding** | | **Studies contributing to the review finding** | **CERQual assessment of confidence in the evidence** | **Explanation of CERQual assessment** | |
| --- | --- | --- | --- | --- | --- |
| **Finding 1: Experiencing inadequate support from husbands/partners was stressful for women and they expressed a need for greater support** | | **Atif 2020, Ayers 2019, Chang 2015, Copeland 2019, Rosario 2016** | High confidence | **Adequacy**  Very minor concerns  **Relevance**  Minor concerns regarding relevance as studies had moderate geographical spread  Studies included both pregnant women and mothers up to one year  **Cohesion**  No concerns  **Methodological limitations**  Minor concerns in 5 studies | |
| **Finding 2:**  **Peer support was helpful to reduce women’s feelings of stress and anxiety, particularly from women who were or had been pregnant themselves** | | **Evans 2017, Harrison 2020, Chang 2015** | Moderate confidence | **Adequacy**  Moderate concerns (only three studies with relatively thin data)  **Relevance**  Minor concerns as studies had moderate geographical spread  Studies included pregnant women and mothers  **Cohesion**  Very minor concerns  **Methodological limitations**  Minor concerns in three studies | |
| **Finding 3: Women valued support from their families but felt stressed when they were being constantly told what to do** | | **Affonso 1993, Atif 2020, Ayers 2019, Bloom 2012, Chang 2015, Copeland 2019, Harrison 2020, Razurel 2011,** | High confidence | **Adequacy**  Very minor concerns (high number of studies included)  **Relevance**  Very minor concerns regarding relevance as studies had moderate geographical spread  Both pregnant women and mother included across the studies  **Cohesion**  No concerns  **Methodological limitations**  Minor concerns in seven studies | |
| **Women’s experience of healthcare: Summary of Review finding** | **Studies contributing to the review finding** | | **CERQual assessment of confidence in the evidence** | **Explanation of CERQual assessment** |  |
| **Finding 4: Women’s experience of unsatisfactory care and poor support from healthcare professionals contributed to stress and anxiety** | **Affonso 1993, Arfaie 2017, Ayers 2019, Bloom 2012, Evans 2017, Harrison 2020, Razurel 2011, Rosario 2017** | | High confidence | **Relevance**  Minor concerns regarding relevance as studies had moderate geographical spread  Studies included both pregnant women and mothers  **Adequacy**  Some individual minor concerns but high number of studies included  **Cohesion**  No concerns  **Methodological limitations**  Minor concerns in seven studies |  |
| **Finding 5: Women expressed frustration and dissatisfaction with the quality of healthcare services available and felt facilities and services were inadequate both during their pregnancy and in the postpartum period** | **Arfaie 2017, Atif 2020, Evans 2017, Harrison 2020** | | High confidence | **Relevance**  Moderate concerns regarding relevance as studies had moderate geographical spread  Studies included pregnant women and mothers  **Adequacy**  Minor concerns  **Cohesion**  No concerns  **Methodological limitations**  Minor concerns in three studies |  |
| **Finding 6: Women found the experience of childbirth anxiety provoking if they felt they had no sense of control or certainty until the baby was born** | **Arfaie 2017, Atif 2020, Ayers 2019, Evans 2017** | | High confidence | **Relevance**  Moderate concerns regarding relevance as studies had moderate geographical spread  Both pregnant women and mothers included  **Adequacy**  Minor concerns  **Cohesion**  No concerns  **Methodological limitations**  Minor concerns |  |
| **Factors that impact on coping: Summary of Review finding** | **Studies contributing to the review finding** | | **CERQual assessment of confidence in the evidence** | **Explanation of CERQual assessment** |  |
| **Finding 7: Faith was recognised as an important method of coping as turning to religion and prayers eased women’s symptoms of stress and anxiety throughout pregnancy and the postpartum period** | **Atif 2020, Bloom 2012, Rosario 2017** | | Moderate confidence | **Relevance**  Minor concerns regarding relevance as studies included a range of geographical locations  Studies mainly included pregnant women  **Adequacy**  Moderate concerns (only three studies with thin data available)  **Cohesion**  No concerns  **Methodological limitations**  Minor concerns |  |
| **Finding 8: Women used a wide range of behavioural strategies to help cope with their symptoms of stress and anxiety including talking which helped women share their concerns and worries** | **Affonso 1993, Atif 2020, Bloom 2012, Chang 2014, Evans 2017, Harrison 2020, Razurel 2011, Rosario 2017** | | High confidence | **Relevance**  Minor concerns as studies included a moderate geographical spread  Both pregnant women and mothers included  **Adequacy**  Minor concerns in only one study  **Cohesion**  No concerns  **Methodological limitations**  Minor concerns in seven studies |  |
| **Finding 9:** **Women experienced stress and anxiety from conflicting, confusing and inconsistent information** | **Harrison 2020, Rowe 2015** | | Low confidence | **Relevance**  Minor concerns regarding relevance as studies had a moderate geographical spread  Included both pregnant women and mothers  **Adequacy**  Serious concerns (only two studies offering thin data)  **Cohesion**  Very minor concerns  **Methodological limitations**  Minor concerns |  |
| **Social Norms and Expectations: Summary of review finding** | **Studies contributing to the review finding** | | **CERQual assessment of confidence in the evidence** | **Explanation of CERQual assessment** |  |
| **Finding 10: Women felt under significant pressure to adhere to perceived social norms and spoke about feelings of judgement** | **Affonso 1993, Atif 2020, Ayers 2019, Chang 2014, Copeland 2019, Harrison 2020, Razurel 2011, Rosario 2017, Rowe 2015** | | High confidence | **Relevance**  Minor concerns  **Adequacy**  No concerns  **Cohesion**  No concerns  **Methodological limitations**  Minor concerns in eight studies |  |
| **Finding 11: Mental health norms and the stigma about perinatal mental illnesses were a barrier to seeking help as women felt embarrassed and ashamed of their mental health difficulties** | **Atif 2020, Harrison 2020, Rowe 2015** | | High confidence | **Relevance**  Minor concerns  **Adequacy**  Minor concerns  **Cohesion**  Very minor concerns  **Methodological limitations**  Minor concerns in two studies |  |
| **Finding 12: Adjusting to life with a new baby including new roles and responsibilities related to being a mother, a partner and being in work, was experienced as stressful** | **Affonso 1993, Atif 2020, Ayers 2019, Chang 2014, Copeland 2019, Harrison 2020, Razurel 2011, Rowe 2015, Rosario 2017** | | High confidence | **Relevance**  Some individual minor concerns but high number of studies included.  **Adequacy**  Very minor concerns  **Cohesion**  No concerns  **Methodological limitations**  Minor concerns |  |
| **Mother and baby’s health: Summary of review finding** | **Studies contributing to the review finding** | | **CERQual assessment of confidence in the evidence** | **Explanation of CERQual assessment** |  |
| **Finding 13: Women encountered a range of health problems during the perinatal period and especially in relation to ‘self- care’ issues** | **Ayers 2020, Copeland 2019, Rosario 2017** | | Moderate confidence | **Relevance**  Minor concerns regarding relevance as studies included a moderate geographical spread  Included both pregnant women and postpartum women  **Adequacy**  Moderate concerns (only three studies with limited data)  **Cohesion**  Minor concerns  **Methodological limitations**  Minor concerns in three studies |  |
| **Finding 14: Women had fears and concerns over the health of their unborn baby and particularly worried that something will go wrong** | **Arfaie 2017, Rowe 2015, Stevenson 2016** | | Moderate confidence | **Relevance**  Minor concerns regarding relevance as studies included a moderate geographical spread  Studies included both pregnant women and mothers  **Adequacy**  Minor to moderate concerns  **Cohesion**  Very minor concerns  **Methodological limitations**  Minor concerns in three studies |  |
| **Finding 15: Infant health problems were a source of stress for women with many women reporting that they were struggling to cope with their baby’s health problems leading to feelings of hopelessness and despair** | **Ayers 2019** | | Low confidence | **Relevance**  Serious concerns regarding relevance as studies included a limited geographical spread  Study only included postpartum women  **Adequacy**  Serious concerns (only one study with thin data)  **Cohesion**  Minor concerns  **Methodological limitations**  Minor concerns |  |
